# Data-driven biological network alignment that uses topological, sequence, and functional information

**DOI:** 10.1186/s12859-021-03971-6

**Published:** 2021-01-29

**Authors:** Shawn Gu, Tijana Milenković

**Affiliations:** Department of Computer Science and Engineering, Eck Institute for Global Health, Center for Network and Data Science, University of Notre Dame, Notre Dame, IN 46556 USA

**Keywords:** Biological networks, Network alignment, Across-species protein functional prediction

## Abstract

**Background:**

Network alignment (NA) can transfer functional knowledge between species’ conserved biological network regions. Traditional NA assumes that it is topological similarity (isomorphic-like matching) between network regions that corresponds to the regions’ functional relatedness. However, we recently found that functionally unrelated proteins are as topologically similar as functionally related proteins. So, we redefined NA as a data-driven method called TARA, which learns from network and protein functional data what kind of topological *relatedness* (rather than similarity) between proteins corresponds to their functional relatedness. TARA used topological information (within each network) but not sequence information (between proteins across networks). Yet, TARA yielded higher protein functional prediction accuracy than existing NA methods, even those that used both topological and sequence information.

**Results:**

Here, we propose TARA++ that is also data-driven, like TARA and unlike other existing methods, but that uses across-network sequence information on top of within-network topological information, unlike TARA. To deal with the within-and-across-network analysis, we adapt social network embedding to the problem of biological NA. TARA++ outperforms protein functional prediction accuracy of existing methods.

**Conclusions:**

As such, combining research knowledge from different domains is promising. Overall, improvements in protein functional prediction have biomedical implications, for example allowing researchers to better understand how cancer progresses or how humans age.

## Background

### Introduction

Many proteins remain functionally unannotated. For example, it has been estimated that 1,936 (29%) of *S. cerevisiae* proteins and 6,612 (33%) of *H. sapiens* proteins remain functionally unannotated [1]. A popular way to uncover missing annotations is to transfer functional knowledge across proteins of different species. This task of *across-species* protein functional prediction is the focus of this paper. The orthogonal task of *within-a-species* protein functional prediction, where a function of a protein in a species is predicted from function(s) of other protein(s) in *the same* species [2], is out of the scope.

Genomic and proteomic sequence alignment (henceforth referred to simply as “sequence alignment”) are commonly used for the task of across-species protein functional prediction. Namely, the goal of sequence alignment is to identify regions of similarity between compared sequences that likely arise from functional or evolutionary relationships between the sequences, which is why numerous studies have relied on the assumption that sequence-similar genes/proteins perform similar functions [2–4]. (Henceforth, we refer to genes and their protein products simply as proteins.) Consequently, such studies have transferred function from an annotated protein to an unnanotated protein if the two are sequence-similar [2–4]. However, many sequence-similar proteins do *not* perform the same function(s), i.e., are functionally unrelated, and many sequence-dissimilar proteins are functionally related [5]; by “functionally related”, we mean that, according to annotation data from Gene Ontology (GO) [6], proteins share at least *k* GO terms (typically, *k* is from 1 to 3), and by “functionally unrelated”, we mean that proteins share no GO terms [5, 7]. For example, of all yeast-human sequence orthologs from YeastMine [8], $\sim 42\%$ are *not* functionally related [5]. Such discrepancy between sequence similarity and functional relatedness is because sequence alone does not dictate a protein’s function. Instead, proteins interact with each other in complex networked ways to carry out cellular functioning. So, we argue that accounting for protein-protein interactions (PPIs) in addition to sequence information will improve functional prediction across species.

Luckily, large amounts of PPI network data are available [9]. Hence, network alignment (NA) can be used to compare PPI networks of different species, in order to find a “good” mapping between their nodes (proteins), i.e., a node mapping that uncovers regions of high network topological (and often sequence) conservation between the species; conservation typically means similarity. So, analogous to sequence alignment, NA can be used to transfer functional knowledge between conserved (aligned) PPI network, rather than just sequence, regions of different species [10–14]. While we focus on computational biology, NA is also applicable to many other domains [12].

NA can be categorized into several broad types, whose high-level input/output/goal differences are as follows (more detailed algorithmic differences between specific NA methods are discussed in section “Methods – Description of existing NA methods”). First, NA can be pairwise (aligns two networks) or multiple (aligns three or more networks) [10, 14]. We focus on pairwise NA because current multiple NA is more computationally complex [15] and generally less accurate [16] than existing pairwise NA. Second, NA can be local or global [11, 14], like sequence alignment. Local NA aims to find highly conserved network regions but usually results in such regions being small. Global NA aims to maximize overall network similarity; while it usually results in large aligned network regions, these regions are suboptimally conserved. Both have their own (dis)advantages [11, 14]. (More on local versus global NA follows shortly.) Third, NA can be one-to-one (each node can be aligned to exactly one distinct node in another network) or many-to-many (a node can be aligned to more than one node in another network).

Traditionally, given networks $G_1(V_1,E_1)$ and $G_2(V_2,E_2)$, local NA has meant the same as many-to-many NA: a relation $R \subseteq V_1 \times V_2$. Also, global NA has meant the same as one-to-one NA: an injective function $f: V_1 \rightarrow V_2$. Over time, local one-to-one and global many-to-many NA methods have been proposed [11]. So, both local and global NA are now $R \subseteq V_1 \times V_2$. The two differ in how many nodes are covered by the aligned node pairs in *R*—much fewer for local than global NA. As global NA has received more attention recently than local NA [10, 14], we focus on global NA. Both one-to-one and many-to-many alignments can be used in our considered task of across-species protein functional prediction. Yet, it is many-to-many NA methods that are the state-of-the-art in this task, which is why our considered methods happen to be many-to-many.

Fourth, three NA method groups exist based on how input data are processed (Table 1 and section “Methods – Description of existing NA methods”).

**Table 1:** Three NA method groups based on how input data are processed

NA method group	Description
Within-network-only	Given two PPI networks, each node’s feature is calculated using only the topological information within the given node’s own network, hence the group name. The nodes’ topological features, which summarize the nodes’ extended PPI network neighborhoods, are then used in various alignment processes (section “Methods – Description of existing NA methods”). For state-of-the-art NA methods from this group, the topological features are based on graphlets [17], which are subgraphs, i.e., small building blocks of networks.
Isolated-within-and-across-network	Given two PPI networks and also sequence information for nodes across networks, each node’s topological feature is calculated in the same way as by within-network-only methods, and *only afterwards* is the sequence information combined with the topological features. The group name comes from the fact that both within-network topological and across-network sequence information are used, but the two are initially processed in isolation from each other and are combined only after the fact. Then, the combined data are used in various alignment processes (section “Methods – Description of existing NA methods”). Note that within-network-only methods can easily be used as isolated-within-and-across-network methods when sequence information is available; the latter lead to better alignments than the former [11].
Integrated-within-and-across-network	Given two PPI networks and sequence information for nodes across networks, the two networks are first “integrated” into one by adding across-network “anchor” links (edges) between the highly sequence-similar proteins and *only then* is any feature extraction or alignment done. So, the third group uses both within-network topological and across-network sequence information. But, they first integrate the two data types and only then process them, hence the group name.

### Motivation

Regardless of which NA category they belong to, almost all existing NA methods assume that it is topological similarity between nodes (i.e., a high level of isomorphism-like matching between their extended PPI network neighborhoods as captured by the nodes’ topological features) that corresponds to the nodes’ functional relatedness, and thus they try to align topologically similar nodes. However, multiple studies observed that while existing NA methods yield high topological alignment quality (many edges are conserved, i.e., the aligned network regions indeed have a high level of isomorphism-like match), their functional alignment quality is far from perfect (often, the aligned nodes are *not* functionally related) [11, 13, 14].

Recently, we attempted to understand this observation [5]. Namely, we questioned the key assumption of current NA—that topologically similar nodes correspond to functionally related nodes. We found for both synthetic and PPI networks that no matter which topological similarity measure was used, the topological similarity of the functionally related nodes was barely higher than the topological similarity of the functionally unrelated nodes [5].

**Fig. 1:**
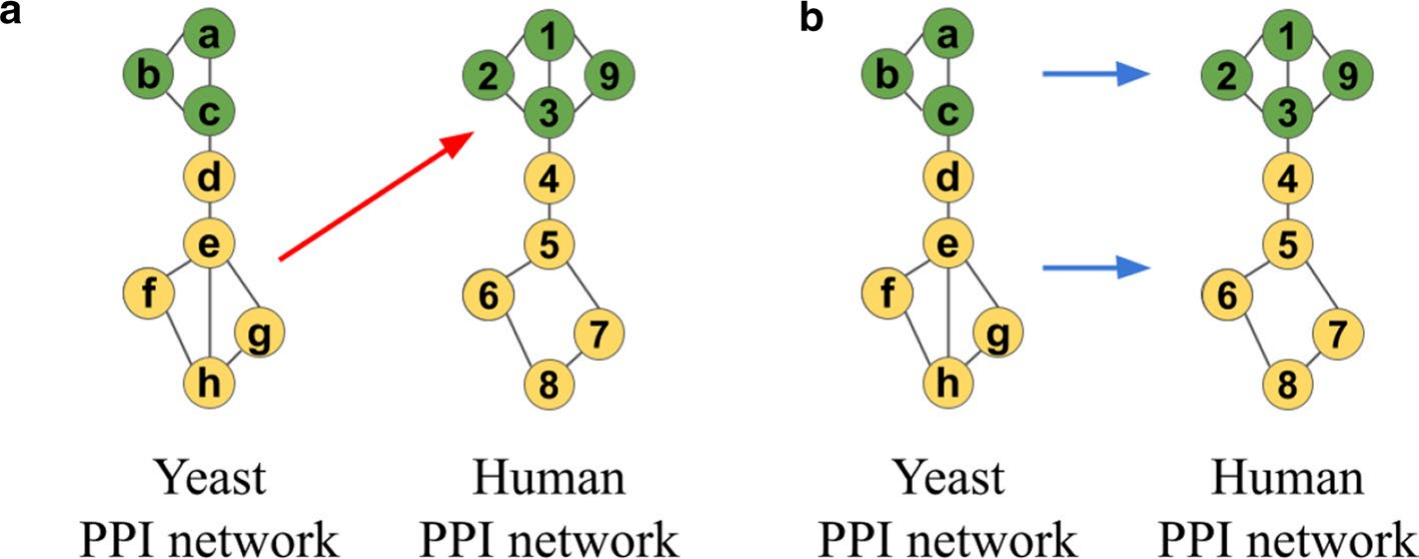
Illustration of topological similarity versus relatedness. Suppose that: (i) PPI networks of yeast and human are being aligned, (ii) the toy networks shown are parts of the full networks, (iii) each node performs either the “green” or “yellow” function, and (iv) because of incompleteness/noisiness of PPI network data or molecular evolutionary events such as gene mutation, duplication or deletion the green functional module in human (nodes 1, 2, 3, and 9) has an extra protein compared to the green module in yeast (nodes *a*, *b*, and *c*), and the yellow module in yeast has an extra interaction compared to the yellow module in human. **a** A topological similarity-based NA method will align yellow nodes *e*, *f*, *g*, and *h* in yeast to green nodes 1, 2, 3, and 9 in human, because both node sets form the same subgraph—a square with a diagonal, i.e., because the set of yellow nodes in yeast are topologically more similar to the set of green nodes in human than to the set of yellow nodes in human. However, this alignment is functionally incorrect because yellow and green nodes perform different functions. **b** Instead, our NA framework based on topological relatedness will use the topological and functional data to learn that a triangle in yeast (*a*, *b*, and *c*) should be aligned to a square-with-diagonal in human (1, 2, 3, and 9) because both perform the same function (green), and that a square-with-diagonal in yeast (*e*, *f*, *g*, and *h*) should be aligned to a square in human (5, 6, 7, and 8) because both perform the same function (yellow). Then, in other parts of the networks, our framework will try to align these learned patterns, to transfer knowledge between them

This shocking result—the current NA assumption failing—led us to redefine the NA problem as a data-driven, i.e., supervised, framework, which learns from PPI network and protein functional data the relationship between proteins’ “topological relatedness” and their functional relatedness, without assuming that topological relatedness means topological similarity. To better understand this framework, we illustrate topological similarity versus topological relatedness in Fig. 1. Loosely speaking, topological relatedness aims to account for data noisiness/incompleteness, evolutionary events, or other, yet-to-be-discovered factors that are likely to break the isomorphism-like assumption of the traditional topological similarity-based NA.

We named our topological relatedness-based NA framework TARA (data-driven NA) [5]. TARA uses supervised classification to learn what topological patterns should be aligned to each other. Given (i) a set of node pairs across the networks being aligned, such that the nodes in a given pair are functionally related, (ii) a set of node pairs across the networks such that nodes in a given pair are not functionally related, and (iii) graphlet-based network topological features of each node pair, TARA divides the node pairs into training and testing data. Then, it uses a classifier to learn from the training data what graphlet features distinguish between the functionally related and functionally unrelated node pairs. Next, given node pairs from the testing data and their graphlet features, TARA predicts whether the nodes in a given pair are functionally related or unrelated. Node pairs predicted as functionally related are added to TARA’s alignment, and this alignment is given to an established across-species protein functional prediction methodology [11] to obtain a list of protein functional annotations (i.e., protein-GO term pairs).

By learning topological relatedness patterns, TARA outperformed, in the task of across-species protein functional prediction between yeast and human, three state-of-the-art NA methods, WAVE [18], SANA [19], and PrimAlign [20]. To better understand the implications of these results, it is important to understand how each method works (Table 2). TARA, WAVE, and SANA are all within-network-only methods. They also all use graphlet-based topological node features. Their key difference is that TARA is supervised, ie., it uses topological relatedness, while WAVE and SANA are unsupervised, i.e., they use topological similarity. Thus, WAVE and SANA were the most fairly comparable methods to TARA. So, we could fairly evaluate whether moving from WAVE’s and SANA’s topological similarity to TARA’s supervision-based topological relatedness helped. TARA significantly outperformed WAVE and SANA, so we could conclude that it did help. PrimAlign is one of very few existing integrated-within-and-across-network methods. Because PrimAlign was already shown to outperform many isolated-within-and-across-network methods [20] on the *exact same data* as in TARA’s evaluation [5], there was no need to evaluate TARA against any methods of that type. Importantly, TARA still outperformed PrimAlign, despite the former being a within-network-only method and hence not using any sequence information, unlike the latter. This already showed how powerful the supervised NA paradigm is. In this study, we push the boundary further. TARA “only” showed that going from unsupervised to supervised for within-network-only methods improved alignment accuracy. But, we already know that going from within-network-only to isolated-within-and-across-network in the unsupervised context improves accuracy [11], and that going from isolated-within-and-across-network to integrated-within-and-across-network in the unsupervised context further improves accuracy [20]. So, a method that is both supervised and of the integrated-within-and-across-network type should be the “best of both worlds”. Thus, here, we propose the first ever method of this type.

**Table 2:** Categories that relevant NA methods belong to

NA method	Method group	Feature type	(Un)supervised?
WAVE	Within-network-only	Topology (graphlets)	Unsupervised
SANA	Within-network-only	Topology (graphlets)	Unsupervised
PrimAlign	Integrated-within- and-across-network	Topology (PageRank-like) and sequence	Unsupervised
TARA	Within-network-only	Topology (graphlets)	Supervised
TARA-TS	Integrated-within- and-across-network	Topology (graphlets) and sequence	Supervised
TARA++	N/A (TARA++ is the overlap of TARA’s and TARA-TS’s predicted protein-GO term annotations)		

### Our contribution

**Fig. 2:**
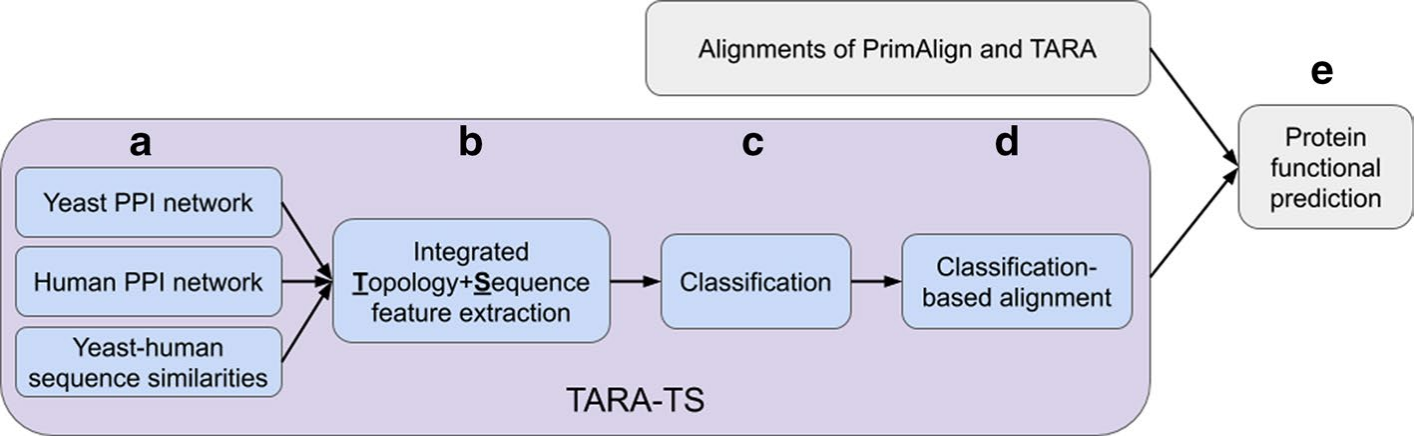
Summary of TARA-TS and our evaluation framework. **a** TARA-TS aims to align two networks (in our study, yeast and human PPI networks). Besides the networks, TARA-TS also uses sequence similar yeast-human protein pairs as anchor links. See section “Methods – Data”. **b** From the networks and anchor links, TARA-TS builds an integrated yeast-human network and extracts integrated topology- and sequence-based features of node (protein) pairs. See section Methods – TARA-TS’s feature extraction methodology”. **c** Given the features, TARA-TS trains a classifier on a training set to learn what features distinguish between functionally related and functionally unrelated node pairs, and then the classifier is evaluated on a testing set. To perform this classification, yeast-human node pairs are labeled. If the two nodes in a given pair are functionally related (intuitively, share GO terms), they are labeled with the positive class; if they are functionally unrelated, they are labeled with the negative class. See section “Methods – Data”. Then, the set of labeled node pairs is split into training and testing sets to perform the classification. Only if classification accuracy is high, i.e., if TARA-TS accurately predicts functionally (un)related nodes to be functionally (un)related, does it make sense to use TARA-TS to create an alignment for protein functional prediction. **d** Node pairs from the testing set that are predicted as functionally related are taken as TARA-TS’s alignment. Note that relying on testing data only to create an alignment avoids any circular argument. See section Methods – TARA-TS’s feature extraction methodology”. **e** Any alignment, of TARA-TS or an existing NA method such as PrimAlign and TARA, can be given to a protein functional prediction framework to predict protein-GO term annotations. Then, the different methods’ alignments are evaluated in terms of their prediction accuracy (we also evaluate their running times). See section “Methods – Using an alignment for protein functional prediction”

We first introduce TARA-TS (TARA within-network Topology and across-network Sequence information) as a novel method implementing the above idea. Then, for reasons discussed below, we integrate TARA and TARA-TS into our final method, TARA++. Figure 2 summarizes key ideas behind TARA-TS and our evaluation framework.

Like TARA, TARA-TS is supervised. Unlike TARA and like PrimAlign, TARA-TS extracts features from an integrated yeast-human network. As a solution to feature extraction, we leverage the extensive research on graph representation learning [21], which embeds nodes of a network into a low dimensional space such that network structure is preserved; the low-dimensional node representations are then used as node features. Network embedding has primarily been studied on the methodological side in the domains of graph theory and data mining/machine learning, and on the application side in the domain of social networks [21–23]. So, given recently recognized promise of network embedding in the domain of computational biology [24], we apply it to this domain. Namely, TARA-TS generalizes a prominent network embedding method that was proposed for within-a-single-network machine learning tasks such as node classification, clustering, and link prediction, to the across-network task of biological NA. Given the node features extracted by network embedding, TARA-TS works just as TARA to produce an alignment. Then, we use this alignment for across-species protein functional prediction.

We compare prediction accuracy of TARA-TS (pairwise, global, many-to-many, integrated-within-and-across-network, supervised) with accuracies of TARA and PrimAlign, as they are state-of-the-art NA methods that were already shown to outperform many other existing NA methods on the *exact same data* as what we use here. So, by transitivity, if TARA-TS is shown to be superior to TARA and PrimAlign, this will mean that TARA-TS is superior to the other existing methods as well. Also, of all existing methods, TARA and PrimAlign are the most similar and thus fairly comparable to TARA-TS. Namely, TARA is pairwise, global, many-to-many, and supervised, like TARA-TS. The difference is that TARA is a within-network-only method while TARA-TS is an integrated-within-and-across-network method (Table 2). PrimAlign is a pairwise, global, many-to-many, and integrated-within-and-across-network method, like TARA-TS. The difference is that PrimAlign is unsupervised while TARA-TS is supervised (Table 2). So, we can fairly test the effect of going from unsupervised to supervised for integrated-within-and-across-network methods.

**Fig. 3:**
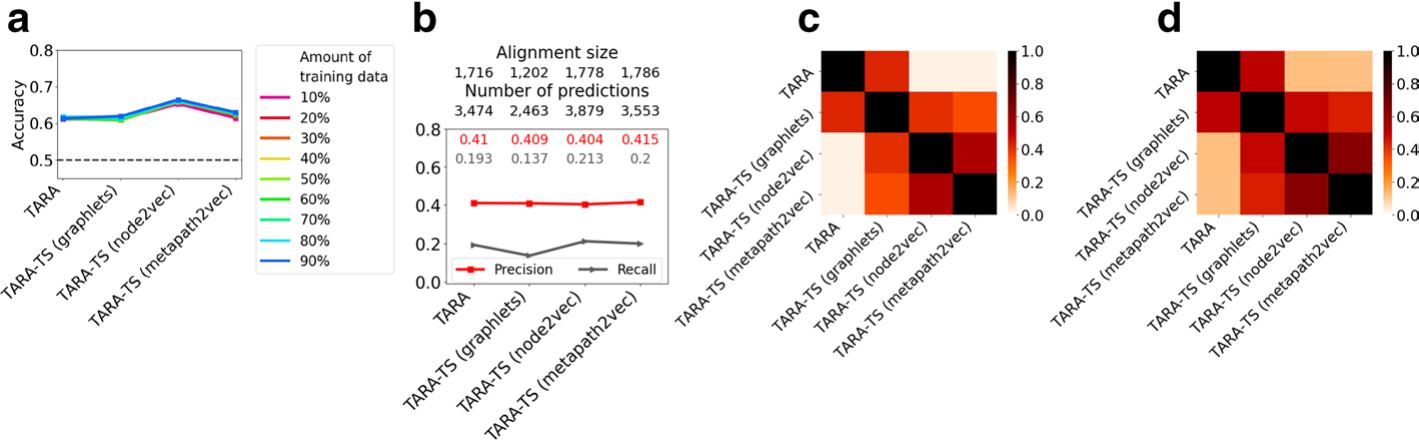
Comparison of the three TARA-TS versions and TARA. Comparison of the three TARA-TS versions and TARA for GO term rarity threshold 25 and ground truth dataset atleast1-EXP, in terms of: **a** classification accuracy, **b** protein functional prediction accuracy, **)** overlap between aligned yeast-human protein pairs, and **d** overlap between predicted protein-GO term associations. In panel (b), the alignment for e.g., TARA contains 1,716 aligned protein pairs and predicts 3474 protein-GO term associations. In panels (c)–(d), the pairwise overlaps are measured via the Jaccard index. Panel (a) encompasses all *y* percent training tests. Panels (b)–(d) are for the 90% training test. Comparisons of different metapath choices for metapath2vec can be found in Additional file 1: Fig. S1. Results for the other ground-truth rarity datasets and percent training tests are shown in Additional file 1: Figs. S2–S8

When we compare TARA-TS against TARA, we actually compare whether using across-network sequence information on top of within-network topological information leads to more accurate predictions, as we expect. Surprisingly, we find that TARA-TS and TARA are almost equally as accurate. Closer examination reveals that their quantitatively similar results are *not* because the two methods are predicting the same information (which would make one of them redundant). Instead, their predicted protein functional annotations are quite complementary. So, we then look at those predictions (protein-GO term associations) that are made by both methods, only those predictions made by TARA-TS but not TARA, and only those predictions made by TARA but not TARA-TS. We find the former (the overlapping predictions) to be more accurate than the predictions made by any one of TARA-TS or TARA alone. Thus, we take this overlapping version of TARA-TS and TARA as our final method, TARA++. In a sense, TARA++ is integrating state-of-the-art research knowledge across computational biology and social network domains, by combining TARA’s graphlet-based topology-only features with TARA-TS’s embedding-based topology-and-sequence features, each of which boosts the other’s performance. Very few studies have explored such a promising direction to date [24]. Importantly, we find that TARA++ not only outperforms TARA but also PrimAlign.

## Methods

### Data

As typically done in NA studies, we analyze yeast and human PPI networks. We consider the exact same PPI networks of yeast (5,926 nodes and 88,779 edges) and human (15,848 nodes and 269,120 edges) that were analyzed and publicly provided by the authors of the PrimAlign study [20]. These networks were also used in the TARA study [5]. All of this allows us to fairly compare results across all of the methods. The two networks contain only physical PPIs, without multi-edges or self-loops.

**Fig. 4:**
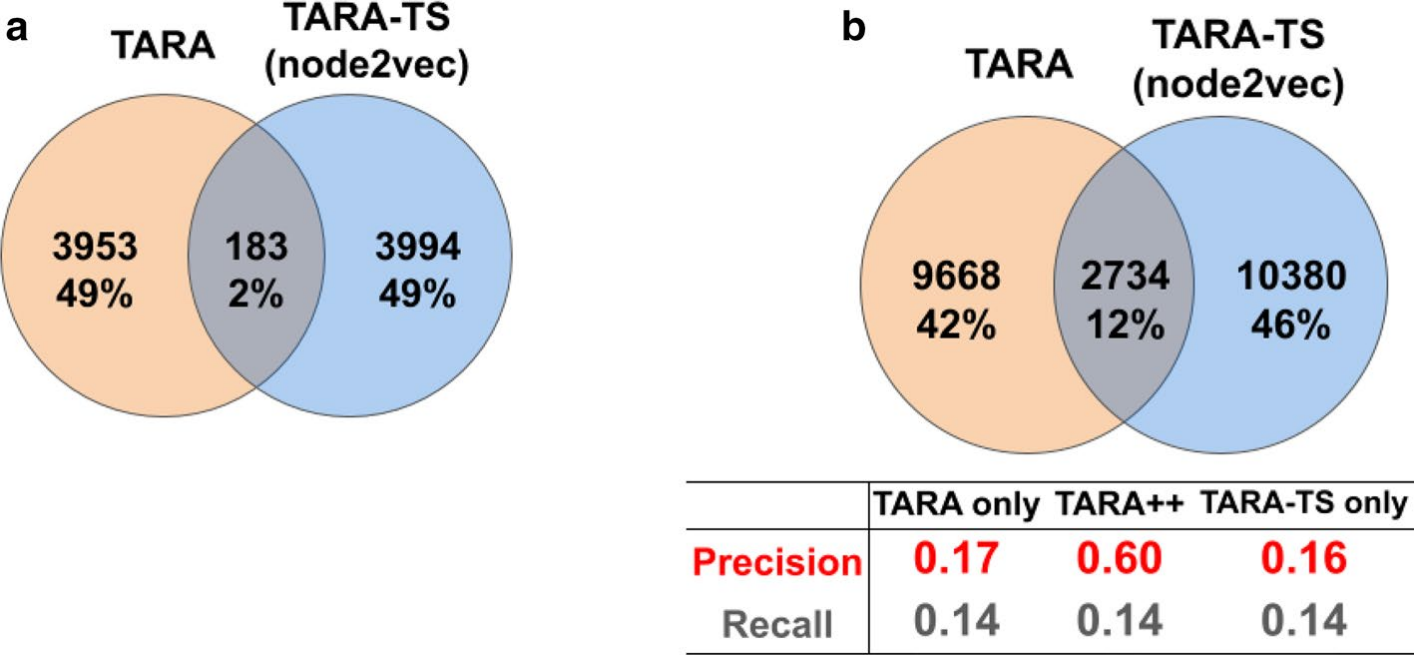
Comparison of TARA-TS and TARA in terms of their alignment and prediction overlaps. Comparison of the selected TARA-TS version and TARA for GO term rarity threshold 50, ground truth dataset atleast1-EXP, and the 90% training test, in terms of overlap between their: **a** aligned yeast-human protein pairs and **b** predicted protein-GO term associations. In panel (b), precision and recall are shown for each of the three prediction sets captured by the Venn diagram; TARA++’s predictions are those in the overlap. The overlaps are for one of the 10 balanced datasets; so, the alignment size and prediction number of a method may differ from those in Fig. 3b, where the statistics are averaged over all balanced datasets. Results for the other ground truth-rarity datasets are shown in Additional file 1: Figs. S9–S10

Similarly, as anchor links between proteins across the networks, we use the exact same 55,594 yeast-human sequance-similar protein pairs that were analyzed and publicly provided by the authors of the PrimAlign study [20]. These had been produced as follows [25]. All-versus-all sequence comparison using BLASTP [26] was performed on human, mouse, fruit fly, worm, and yeast. Only protein pairs with E-value sequence similarities $\le 10^{-7}$ had been kept for further consideration, which yielded 55,594 yeast-human protein pairs with such E-values.

Our supervised NA framework requires knowledge about whether two proteins are functionally related. We next outline the procedure for determining functional relatedness, which mirrors the steps from our past TARA study [5]. As typically done, we define functional relatedness using GO annotation data (from August 2019). Considering biological process GO terms and experimentally inferred protein-GO term annotations (evidence codes EXP, IDA, IPI, IMP, IGI, or IEP), if at least *k* GO terms are shared between a yeast protein and a human protein, we define that protein pair as functionally related. We vary *k* from 1 to 3. These are values of *k* that are typically analyzed, because even in unsupervised and especially in supervised NA studies, larger values of *k* result in insufficiently many pairs of functionally related nodes [5, 7]. Regardless of the *k* value, we define a protein pair as functionally unrelated if the two proteins share no GO terms *of any kind*. This gives the *atleast1-EXP*, *atleast2-EXP*, and *atleast3-EXP* ground truth datasets.

Traditionally, NA studies have considered all GO terms available in a given ground truth dataset. However, it is well known that not all GO terms are “created equally”, meaning that a GO term that is more general and thus higher in the GO tree hierarchy is more likely to annotate a given number of proteins compared to a more specific GO term that is lower in the hierarchy. This is why it might be worth considering only specific-enough GO terms. As a way to deal with this in the context of NA, recent work proposed accounting for the frequency of GO terms (for a given GO term, the number of proteins in the data under consideration that are annotated by that term) [27]. Indeed, in our TARA study, we found that considering rarer (i.e., more specific) GO terms led to higher protein functional prediction accuracy [5]. So, here, we consider the same three GO term rarity thresholds as in the TARA study: (i) all GO terms (i.e., ALL), which corresponds to traditional NA evaluation, (ii) more specific GO terms that appear 50 times or fewer (i.e., threshold of 50), and (iii) even more specific GO terms that appear 25 times or fewer (i.e., threshold of 25).

**Fig. 5:**
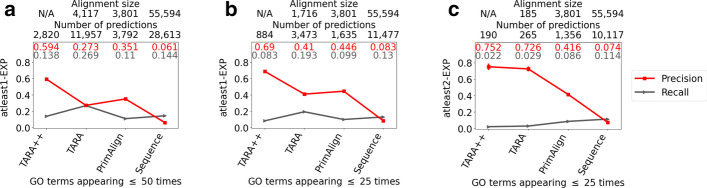
Comparison of TARA++ and three existing methods in the task of protein functional prediction. Comparison of TARA++ and three existing methods in the task of protein functional prediction, for rarity thresholds **a** 50 and **b, c** 25, and for ground truth datasets **a, b** atleast1-EXP and **c** atleast2-EXP. The alignment size (the number of aligned yeast-protein pairs) and number of functional predictions (predicted protein-GO term associations) are shown for each method, except that TARA++ does not have an alignment *per se*. i.e., TARA++ comes from the overlap of *predictions* made by TARA and TARA-TS; hence the “N/A”s. For TARA++ and TARA, results are averages over all balanced datasets; the standard deviations are small and thus invisible. Results for the other ground truth-rarity datasets are shown in Additional file 1: Fig. S11

For a given GO term rarity threshold, all GO terms not satisfying the threshold are filtered out. Then, for each atleast*k*-EXP ground truth dataset, only proteins that share at least *k* GO terms from the remaining list are considered to be functionally related, and still, proteins that share no GO terms, regardless of rarity, are considered to be functionally unrelated. For example, proteins that share at least two (experimentally inferred biological process) GO terms, such that each GO term annotates 25 or fewer proteins, are considered functionally related in the “atleast2-EXP at the 25 GO term rarity threshold” dataset. There is a total of nine such “ground truth-rarity” datasets, resulting from combinations of the three atleast*k*-EXP ground truth datasets and the three GO term rarity thresholds.

### TARA-TS’s feature extraction methodology

TARA-TS needs to extract features that capture both within-network topological and across-network sequence information from the integrated network, which consists of 21,774 nodes (5,926 yeast + 15,848 human proteins) and 413,493 edges (88,779 yeast PPIs + 269,120 human PPIs + 55,594 anchor links). We examine several feature extraction approaches.

First, we use the same graphlet-based feature extraction method as TARA, simply applied to the integrated network rather than the two individual networks; for technical details about the graphlet features that we use, see Additional file 1: Section S1.1.1. In this way, we can test whether going from TARA’s within-network-only approach to TARA-TS’s integrated-within-and-across-network approach improves NA accuracy. We refer to this version of TARA-TS as “TARA-TS (graphlets)”.

Second, we apply a prominent network embedding method based on random walks to the integrated network to extract features, namely node2vec [28]; for technical details about node2vec and why we use node2vec over other network embedding methods, see Additional file 1: Section S1.1.2. We refer to this version of TARA-TS as “TARA-TS (node2vec)”.

Third, node2vec does not capture heterogeneous information in the integrated network, i.e., does not distinguish between different types of nodes (yeast and human) or edges (yeast PPIs, human PPIs, and yeast-human sequence-based anchor links). So, we also test metapath2vec [29], which essentially is node2vec generalized to heterogeneous networks. Intuitively, this approach uses “metapaths” to capture the heterogeneous information, which define the types of nodes that should be visited by random walks; for technical details about metapath2vec, see Additional file 1: Section S1.1.3. We refer to this version as “TARA-TS (metapath2vec)”.

Henceforth, we refer to TARA-TS (graphlets), TARA-TS (node2vec), and TARA-TS (metapath2vec) as different “TARA-TS versions”. If we just say “TARA-TS”, the discussion applies to all three versions.

In theory, the heterogeneous information could be captured not just via metapaths but also via heterogeneous graphlets [30] (versus homogeneous graphlets discussed thus far). However, in practice, heterogeneous graphlet counting is infeasible for as large networks as studied in this paper, due to its exponential computational complexity. This is not an issue for homogeneous graphlet counting because methods such as Orca [31] rely on combinatorics to infer the counts of some (larger) graphlets from the counts of other (smaller) graphlets, significantly reducing the computational complexity. However, no publicly available implementation of combinatorial relationships for counting heterogeneous graphlets exists. Similar holds for a method that *directly* extracts the feature vector of a *node pair* [32], versus extracting graphlet features of *individual nodes* and then combining these, as TARA does: no combinatorial approach for direct node pair graphlets exists. Instead, current heterogeneous and node pair graphlet counting require exhaustive graphlet enumeration and are thus infeasible.

Lastly, we discuss why we do not use feature vectors from PrimAlign, the next most comparable method to TARA [5] that already integrates within-network topological and across-network sequence information. This is because PrimAlign’s algorithmic design does not allow for feature vector extraction. As discussed in more detail in section “Methods – Description of existing NA methods”, PrimAlign models the integrated network as a Markov chain, which is then repeatedly transitioned until convergence. This means that the weights between every node pair are updated at the same time, based on the weights of every node pair from the previous state of the chain. So, PrimAlign operates on every node pair *at once* with respect to their weights, rather than on *individual nodes or node pairs* with respect to any kind of feature vector, meaning that we cannot easily extract such information.

### TARA-TS’s classification and alignment generation

We must first evaluate whether TARA-TS can correctly predict nodes as functionally (un)related. If not, there would be no point to use it to form an alignment. To evaluate this, we train and test a classifier as follows.

**Fig. 6:**
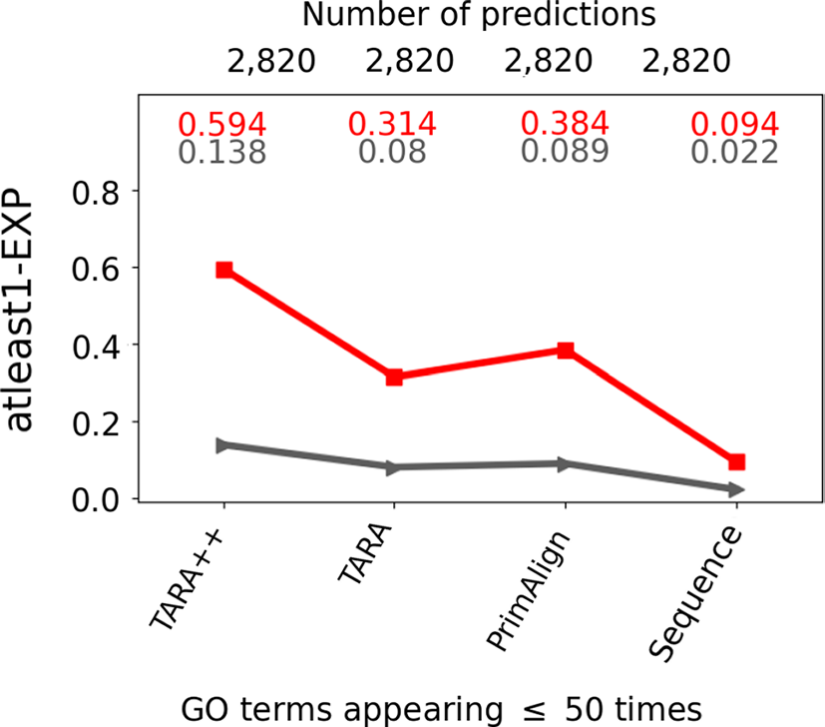
Comparison of TARA++ and three existing methods when all make the same number of predictions. Representative results (for one ground truth-rarity dataset) comparing TARA++ and three existing methods in the same way as in Fig. 5a except that here all methods make the same number of predictions. The remaining results (for the other ground truth-rarity datasets) are shown in Additional file 1: Fig. S12

**Fig. 7:**
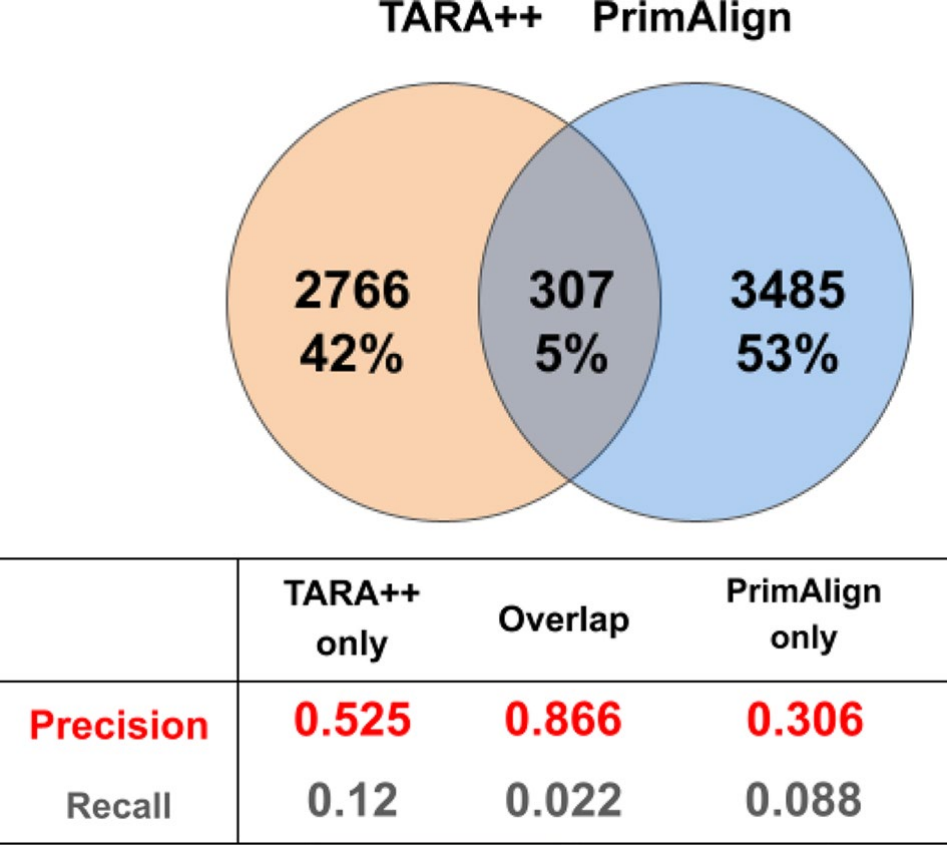
Comparison of TARA++ and PrimAlign in terms of their prediction overlaps. Representative results (for GO term rarity threshold 50 and ground truth dataset atleast1-EXP) comparing TARA++ and PrimAlign in the same way as TARA and TARA-TS are compared in Fig. 4b. The remaining results (for the other ground truth-rarity datasets) are shown in Additional file 1: Fig. S13

**Fig. 8:**
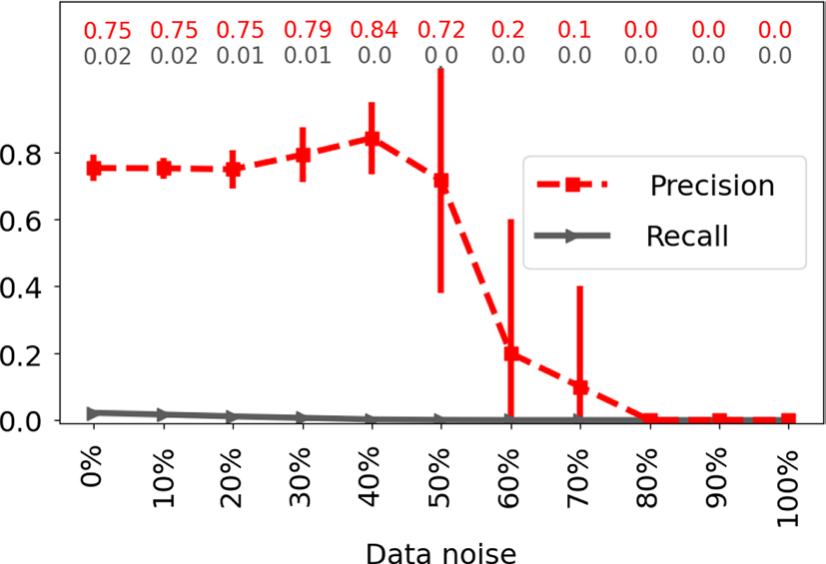
Robustness of TARA++ to data noise. Robustness of TARA++’s protein functional prediction accuracy as data noise increases from 0 to 100%, for GO term rarity threshold 25 and ground truth dataset atleast2-EXP

For a given ground truth-rarity dataset (section “Methods – Data”), the positive class consists of functionally related node pairs, and the negative class consists of functionally unrelated node pairs. Because the latter is much larger, we create a balanced dataset by undersampling the negative class to match the size of the positive class, as typically done [33]. Due to randomness in sampling, we create 10 balanced datasets and repeat the classification process for each, averaging results over them.

For a given balanced dataset, we split it into two sets: *y* percent of the data is randomly sampled and put into one set, and the remaining $(100-y)$ percent is put into the other set. This sampling is done with the constraint that in each of the two sets, 50% of the data instances have the positive class and 50% have the negative class. Again, due to randomness in sampling, we repeat this 10 times to create 10 data splits of $y/(100-y)$ percent and repeat the classification process for each, averaging results over them.

For a given $y/(100-y)$ split, we train a logistic regression classifier on the set containing *y* percent of the data (the training set). We use this trained classifier to predict on the remaining $(100-y)$ percent of the data (the testing set), measuring the accuracy and area under receiver operating characteristic curve (AUROC).

In summary, for a given *y*, for each balanced dataset, we have 10 accuracy and 10 AUROC scores, corresponding to the 10 data splits; for each measure, we compute the average over the 10 splits, obtaining a single accuracy and single AUROC. Then, for a given *y*, given the single accuracy and single AUROC for each balanced dataset, i.e., given 10 accuracy and 10 AUROC scores for the 10 balanced datasets, for each measure, we compute the average over the 10 balanced datasets to obtain a final accuracy and a final AUROC score for that *y*. In our evaluation, we vary *y* from 10 to 90 in increments of 10; each variation is called a “*y* percent training test”. This allows us to test how the amount of training data affects the results, which is important because in many real-world applications, not much data may be available for training.

Only if the average accuracy and AUROC are high, i.e., if TARA-TS accurately predicts functionally (un)related nodes to be functionally (un)related, does it make sense to use TARA-TS to create an alignment for protein functional prediction. If this is the case, we create an alignment as follows. Given one $y/(100-y)$ split and the classifier trained on it, we take every node pair from the testing set that is predicted as functionally related and add it to the alignment. Here, it is important to only use the testing set for the alignment. This way, because there is no overlap between node pairs in the testing set and node pairs in the training set, the alignment will not contain any node pairs that were trained on. Consequently, this avoids a circular argument when constructing TARA-TS’s alignment. For simplicity, we do not repeat this process for all data splits, as we found that the split choice had no major effect on the classification performance. We only use the “first” one, which in our implementation corresponds to a starting seed of 0 for Python’s random number generator when performing sampling. We have a total of 270 alignments, corresponding to all combinations of the 3 TARA-TS versions, the 9 percent training tests, and the 10 balanced datasets.

### Using an alignment for protein functional prediction

An ultimate goal of biological NA is across-species protein functional prediction, so each NA method must be evaluated in this context. We use a (TARA-TS’s or an existing method’s) alignment in an established protein functional prediction framework [11], as follows. Suppose that we are evaluating an alignment for the ground truth-rarity dataset atleast*k*-EXP at the *r* GO term rarity threshold (e.g., atleast2-EXP at the 25 GO term rarity threshold). Let us define “relevant GO terms” as all GO terms in that ground truth-rarity dataset. Then, the framework makes predictions for each protein *u* in the alignment that is annotated by at least *k* relevant GO terms (i.e., for each protein for which a prediction can actually be made at that ground truth-rarity dataset). To do so, first, the framework hides *u*’s true GO term(s). Then, for each relevant GO term *g*, the framework determines if the alignment is significantly “enriched” in *g*. The hypergeometric test is used for this, in order to calculate if the number of aligned node pairs in which the aligned proteins share *g* is significantly high (see below). If so, then node *u* is predicted to be annotated by GO term *g*. Repeating for all applicable proteins and GO terms results in the final list of predicted protein-GO term associations. From this prediction list, the framework calculates the precision (percentage of the predictions that are in a given ground truth-rarity dataset) and recall (percentage of the protein-GO term association from a given ground truth-rarity dataset that are among the predictions).

The hypergeometric test works as follows. If *Y* is the set of all yeast proteins and *H* is the set of all human proteins, then let $M = \{(y, h) \in Y \times H$ | each of *y* and *h* is annotated by at least *k* relevant GO terms$\}$. Let $N = \{(y, h) \in M$ | each of *y* and *h* is annotated by $g\}$. Note that *g* refers to the same GO term as in the previous paragraph. If *A* is the alignment of interest, let $O = \{(y, h) \in A$ | each of *y* and *h* is annotated by at least *k* relevant GO terms$\}$. Finally, let $P = \{(y, h) \in O$ | each of *y* and *h* is annotated by $g\}$. Then, the *p*-value resulting from the hypergeometric test is the probability of seeing |*P*| or more successes (i.e., node pairs that share *g*) if we randomly choose |*O*| elements from *M* given that *M* contains |*N*| successes (for example, in Python, this would correspond to 1 - scipy.stats.hypergeom.cdf($|P|-1$, |*M*|, |*O*|, |*N*|)).

We ensure that there is no circular argument when predicting an annotation between a protein *u* and a GO term *g* from the alignment of interest, even if this particular annotation might have been used to construct the training data. Namely, to predict protein *u* as being annotated by GO term *g*, *u* must have been aligned to some protein *v* that also has GO term *g*, in order for the functional knowledge *g* to be transferred from *v* to *u*. For this to happen, node pair (*u*, *v*) must have appeared in the testing data (and been predicted as functionally related, thus being placed into the alignment). This means that (*u*, *v*) could not have appeared in the training data, because the training and testing data do not overlap (section “Methods – TARA-TS’s classification and alignment generation”). Even if some other node pair (*u*, *w*), where both *u* and *w* are annotated by *g*, appears in the training data, which could happen only if *u* is annotated by *g* and *w* is annotated by *g*, the prediction from the alignment of *u* having *g* could not have originated from the pair (*u*, *w*) that the alignment was trained on. Instead, this prediction must have originated from node pair (*u*, *v*) that is not in the training data. This avoids a circular argument when predicting protein-GO term annotations.

### Description of existing NA methods

Here, we describe existing NA methods to explain why we ultimately compare against TARA and PrimAlign out of all existing methods.

First, we discuss within-network-only and isolated-within-and-across-network methods. They have two parts. First, similarities are computed for all pairs of nodes across networks. For within-network-only methods, these are topological similarities (computed by comparing the nodes’ topological features). For isolated-within-and-across-network methods, these are a weighted sum of the nodes’ topological and sequence similarities. Second, an alignment strategy aims to maximize the total similarity over all aligned nodes while also conserving many edges. Two types of alignment strategies exist. One type is “seed-and-extend”, which progressively builds an alignment by adding to it one node pair at a time. WAVE [18], when paired with graphlet-based topological similarities, is a state-of-the-art method of this type. The other type is a “search algorithm” that optimizes an objective function over the solution space of possible alignments. We pioneered search algorithm-based NA with MAGNA and MAGNA++ [34, 35]. The more recent SANA [19] is a state-of-the-art approach of this type, whose objective function is generally graphlet-based.

Next, we discuss integrated-within-and-across-network NA methods. PrimAlign [20] is a state-of-the-art method of this type. After linking networks being aligned via anchors, PrimAlign creates a Markov chain out of the integrated network, converting the edge weights to transition probabilities (in an unweighted network, the weights are set to 1 before converting to transition probabilities). The chain is then transitioned repeatedly until it converges, which redistributes the across-network node pair scores using a PageRank-like algorithm. Node pairs across networks that are above some threshold are outputted as the alignment.

MUNK also links the original networks via anchors, but it uses matrix factorization to obtain an alignment [36]. In our preliminary analyses, MUNK’s similarity scores could not distinguish between functionally related and functionally unrelated proteins. Furthermore, Nelson et al. [24] found IsoRank [37] to outperform MUNK, despite the former being an early method and the latter a recent method. IsoRank was already outperformed by many NA methods that appeared after it, which in turn were outperformed by WAVE and SANA, which were then outperformed by TARA and PrimAlign (see below). Thus, because we compare against TARA and PrimAlign in this study, there is no need to also compare against MUNK.

Unlike TARA++, the previously mentioned methods do not use functional (GO) information to produce alignments but only to evaluate them. DualAligner [38] does use such information, but not to determine classification labels (“functionally related” and “functionally unrelated”) like TARA++ does. Instead, the method aligns groups of nodes that are all annotated with a given GO term, and then seeds-and-extends around these groups to match proteins that do not have any GO annotations, resulting in the final alignment. We do not consider DualAligner in this study, as it is quite old (from 2014). More recent, state-of-the-art methods have appeared since [10, 14].

The above methods are unsupervised. Many other such methods exist [14]. TARA and PrimAlign, which we consider in this study, already outperformed the other methods, including AlignMCL, AlignNemo, CUFID, HubAlign, IsoRankN, L-GRAAL, MAGNA, MAGNA++, MI-GRAAL, NETAL, NetCoffee, NetworkBLAST, PINALOG, SANA, SMETANA, and WAVE [5, 18–20]. In turn, these outperformed GHOST, IsoRank, NATALIE, PISwap, and SPINAL [19]. This, plus TARA and PrimAlign being the most similar and thus fairly comparable to TARA++, is why we focus on these two existing methods. Also, some supervised methods (besides TARA, already discussed) exist, as follows.

IMAP [39] uses supervised learning differently than TARA++. As input, IMAP requires a starting (unsupervised, topological similarity-based) alignment between two networks; as such, it still suffers from the topological similarity assumption. Then, it obtains graphlet features for node pairs. Node pairs from the starting alignment form the positive class, while the other node pairs are sampled to form the negative class. Then, IMAP trains a linear regression classifier on these two classes. After, this data is “re-classified”, but instead of assigning a class, IMAP assigns a score corresponding to the probability that the two nodes should be aligned. A matching algorithm (e.g., Hungarian) is applied to these scores to form a new alignment, which is then fed back to IMAP. This process iterates while alignment quality improves. We did try to test IMAP. Its code was not available. Our attempts at implementing IMAP ourselves led to significantly worse results than those reported in the IMAP paper. So, we could not consider IMAP in our evaluation.

MEgo2Vec [40], also supervised, is a social NA method for matching user profiles across different online media platforms. Features of user profiles are obtained using graph neural networks and natural language processing techniques, and these are used to train a classifier to predict whether two profiles from different platforms correspond to the same person. A big part of MEgo2Vec is the various natural language processing techniques to match users’ names, affiliations, or research interests, meaning that it cannot be easily applied to PPI networks.

## Results

### Comparison of TARA-TS versions

*Classification.* Here, we study classification performance of the three TARA-TS versions (graphlets, node2vec, metapath2vec) and TARA, i.e., how correctly they predict as functionally (un)related the protein pairs from testing data in a given *y* percent training test. We would ideally do this on all nine ground truth-rarity datasets. However, two of them, atleast3-EXP at the 50 and 25 thresholds, are too small for TARA-TS and TARA to perform any classification on; data scarcity is a general challenge that machine learning methods face though, and not specific to just TARA-TS and TARA. Thus, we have seven viable ground truth-rarity datasets.

Due to space constraints, we discuss the effect of various parameters (*k* in atleast*k*-EXP, GO term rarity threshold, and *y* percent training test) on the classification performance of a given TARA-TS version, for each version, in Additional file 1: Section S2.1. Instead, here we focus on comparing the three TARA-TS versions and TARA.

We expect all TARA-TS versions to have higher accuracy and AUROC than TARA, as they extract topology plus sequence features from the integrated yeast-human network, unlike TARA, which extracts topology features only within each individual network. However, we find that this is not always the case (Fig. 3a and Additional file 1: Figs. S2–S3): (i) The *relative* accuracy change of TARA-TS (graphlets) over TARA ranges from −3% (decrease) to 5% (increase), depending on the $\hbox {atleast}k$-EXP ground truth dataset, GO term rarity threshold, and *y* percent training test, with an average change of 0%; and its relative AUROC change ranges from −3 to 5%, with an average change of 1%. (ii) TARA-TS (node2vec) does always improve over TARA though. Its relative accuracy change over TARA ranges from 6 to 27%, with an average change of 14%; and its relative AUROC change ranges from 9 to 32% with an average change of 16%. (iii) As for TARA-TS (metapath2vec), we also see improvement over TARA, though not as large as for TARA-TS (node2vec). In particular, the relative accuracy change of TARA-TS (metapath2vec) over TARA ranges from −1 to 14% with an average change of 6%; and its relative AUROC change ranges from 2 to 15%, with an average change of 7%.

Overall, we find that in terms of classification performance, TARA-TS (node2vec) performs the best, followed by TARA-TS (metapath2vec), and followed by TARA-TS (graphlets) and TARA that are tied; all four perform significantly better than at random (Additional file 1: Figs. S2–S3).

*Protein functional prediction.* Here, we evaluate the protein functional prediction accuracy of alignments of the three TARA-TS versions and TARA. Per discussion in Additional file 1: Section S2.1, for each of TARA-TS and TARA, different *y* percent training tests have only marginal differences in classification accuracy. For this reason, henceforth, for simplicity, we only consider the 10, 50, and 90 percent training tests; 10 and 90 allow us to test the extremes and 50 allows us to test the middle. Recall that classification cannot be performed on two (small) ground truth-rarity datasets, atleast3-EXP at thresholds 50 and 25, so no alignments exist for them, and thus protein functional prediction is not possible. So, for each TARA-TS version and TARA, we have 21 evaluation tests, resulting from combinations of the seven viable ground truth-rarity datasets and the three selected *y* values.

First, we analyze each TARA-TS version. The following three trends are expected in terms of each version’s performance. (i) Precision will likely increase and recall will likely decrease as the amount of training data goes from 10 to 50 to 90. We expect precision to increase because a classifier trained on a larger dataset will potentially be more accurate. Consequently, the testing dataset will be smaller. So, the alignment produced by a given method version will contain fewer node pairs. This in turn is expected to yield fewer predictions and thus to decrease recall. (ii) Precision will likely increase and recall will likely decrease as the requirement for functional relatedness becomes more stringent, i.e., as the value of *k* in the $\hbox {atleast}k$-EXP ground truth datasets goes up. Namely, increase in precision is expected because at a larger *k* value, training is done on more reliable data. Decrease in recall is expected because at a larger *k* value, there will be less data overall, and hence less testing data. So, a similar argument as in point (i) above applies. (iii) Precision will likely increase and recall will likely decrease as the GO term rarity threshold decreases, i.e., as rarer GO terms are considered. This is based on the observation that rarer GO terms may be more meaningful [5, 27], leading to smaller but higher quality data. As such, higher precision and lower recall are expected for similar reasoning as in points (i) and (ii) above. We find that all three expected trends hold for all TARA-TS versions (Additional file 1: Figs. S4–S6).

Second, we compare the performance of the three TARA-TS versions and TARA. Interestingly, even though TARA-TS (node2vec) has superior classification performance (Fig. 3(a)), all four methods yield almost equal protein functional prediction accuracy (Fig. 3(b) and Additional file 1: Figs. S4–S6). Further unexpected is that TARA-TS has similar accuracy to TARA, despite the former using sequence information that TARA does not. We take a closer look at the alignments and predictions made by each method to see if the different methods are aligning the same nodes, or predicting the same protein-GO term associations. So, we investigate how much their alignments overlap (Fig. 3(c)), and how much their predictions overlap (Fig. 3(d)). We find that the different methods are all aligning and predicting at least somewhat different information from each other. Yet, their predictions are equally accurate. Furthermore, we find that TARA is more similar to (i.e., overlaps the most with) TARA-TS (graphlets) than to TARA-TS (node2vec) and TARA-TS (metapath2vec), which makes sense since the former uses graphlets to extract feature vectors like TARA, and the latter two do not. Moreover, TARA-TS (node2vec) and (metapath2vec) are more similar to each other than to the other methods, which is also expected since they both use a similar random walk-based feature extraction method.

It is surprising that TARA-TS (graphlets) does not improve upon TARA, i.e., that the additional sequence information does not improve upon only topological information. It is also surprising that TARA-TS (metapath2vec) does not improve upon TARA-TS (node2vec) – both use a similar random walk-based embedding process, but metapath2vec additionally accounts for the heterogeneous information in the integrated network. We discuss potential reasons for these two unexpected findings in section “Discussion”.

Because TARA-TS (node2vec) not only yields the best classification performance, predicting functional (un)relatedness the best out of all TARA-TS versions, but also captures the most novel protein functional information compared to TARA (i.e., the predictions it makes overlap the least to those made by TARA out of all TARA-TS versions), we continue only with TARA-TS (node2vec) as the selected TARA-TS version.

### TARA-TS versus TARA in the task of protein functional prediction: toward TARA++

Focusing on TARA-TS (node2vec) as the selected TARA-TS version (i.e., simply as TARA-TS), we zoom into the comparison between it and TARA. The two methods have different alignments and make different predictions (Fig. 4), so how can they still have similar protein functional prediction accuracy? To answer this, we look at the precision and recall of predictions made by both methods, only those predictions made by TARA-TS but not TARA, and only those predictions made by TARA but not TARA-TS (Fig. 4(b) and Additional file 1: Fig. S10). From this, we highlight two findings. First, graphlets, which use only topological information, perform as well as network embedding features that use both topological and sequence information. This is supported by the fact that predictions made only by TARA and only by TARA-TS produce similar accuracy in almost all evaluation tests. Second, predictions made by both methods are significantly more accurate than predictions made by any one method alone. We discuss these findings further in section “Discussion”.

Because the overlap of predictions of TARA-TS and TARA has such high accuracy, we take it as our new TARA++ method, which we consider further. For simplicity, for each of the seven viable ground truth-rarity datasets, we consider either TARA++10, TARA++50, or TARA++90 as a representative percent training test. That is, we pick seven “TARA++ versus existing methods” evaluation tests from the 21 total. We choose the percent training tests that represent TARA++’s best results. Namely, we look for the percent training test with high precision (predictions are accurate) as well as a large number of predictions (maximize the biological knowledge uncovered). So, we choose TARA++90 for all ground truth-rarity datasets except atleast2-EXP at the 50 and 25 rarity thresholds, where we choose TARA++10. Henceforth, we refer to all of the selected TARA++ versions simply as TARA++.

### TARA++ versus existing NA methods in the task of protein functional prediction

*Accuracy.* We compare TARA++’s predictions against those of two most fairly comparable state-of-the-art methods, TARA and PrimAlign. Also, we consider predictions resulting from using only sequence information, Sequence. Here, we treat the 55,594 anchor links by themselves as the alignment; as no topological information is used, this is not an NA method. With TARA and Sequence, we can separately analyze each aspect, i.e., within-network topological information only and across-network sequence information only, and evaluate how each compares to our integrative TARA++. (TARA++’s predictions are by definition a subset of TARA’s predictions, and so we expect TARA++ to have higher precision but lower recall than TARA.) With PrimAlign, we can evaluate how this integrative but unsupervised method compares to our also integrative but supervised TARA++. Importantly, TARA and PrimAlign were already shown to outperform many previous NA methods (section “Methods – Description of existing NA methods”). So, comparing to these two methods is sufficient. Also, keep in mind that like with TARA, a theoretical recall of 1 is not necessarily possible with TARA++. This is because for a given training/testing split, TARA++ uses a part (up to 90%) of the ground truth functional data for training, and so for that split, it is impossible to make predictions for the training data portion, i.e., to transfer functional knowledge from node *u* to node *v* when the node pair (*u*, *v*) is in the training data.

Both precision and recall are important. However, in the biomedical domain, if one has to choose between the two measures, we believe that precision is favored. As an illustration, let us compare the following two scenarios: (i) making 30 predictions of which 27 are correct, i.e., having a small number of mostly correct predictions, and (ii) making 300 predictions of which 100 are correct, i.e., a large number of mostly incorrect predictions. The former, having higher precision but lower recall than the latter, is more viable for potential wet lab validation.

Our key results are as follows (Fig. 5 and Additional file 1: Fig. S11). In terms of precision, TARA++ is the best for 6 out of 7 ground truth-rarity datasets. It is only slightly inferior to PrimAlign for 1 out of 7 datasets (atleast1-EXP for ALL GO terms), but TARA++ has much higher recall than PrimAlign on this dataset. Speaking of recall, TARA is expected to always outperform TARA++, and this is what we observe. Of the remaining existing methods, TARA++ is the best for 2 out of 7 datasets—atleast1-EXP at the ALL and 50 rarity thresholds–even though TARA++ makes much *fewer* predictions than the next best method, Sequence. For the other datasets, TARA++’s recall is lower than that of PrimAlign and Sequence. This is expected, since TARA++ makes fewer predictions than the other methods. Importantly, the difference in recall between TARA++ and every other method is relatively small, for example only 0.06 lower on average compared to TARA, while TARA++ is much better in terms of precision than every other method, for example 0.2 greater on average compared to TARA. As discussed above, such a trade-off between precision and recall is worth it for our task.

We see that the precision of TARA++ is much greater than just the sum of TARA’s and Sequence’s precisions, suggesting that integrating within-network topological and across-network sequence information has compounded effects. This further highlights the need for such approaches.

In the above analyses, we account for the default number of predictions made by each method for the given ground truth-rarity dataset. These numbers do not necessarily match between the different methods. Consequently, TARA++ may have high precision simply because it makes the fewest number of predictions. Nonetheless, when we enforce that each method produces the same number of predictions, we again find that TARA++ is the best of all considered NA methods in a majority of all evaluation tests, in terms of both precision and recall (Fig. 6, Additional file 1: Section S2.2, and Additional file 1: Fig. S12).

Combining TARA and TARA-TS into TARA++ results in such high accuracy. So, we also investigate the overlap between TARA++ and PrimAlign (Fig. 7 and Additional file 1: Fig. S13). The number of overlapping predictions is small, suggesting complementarity between TARA++ and PrimAlign. However, TARA++ still has an advantage when it comes to predicting protein function, as the predictions made only by TARA++ have higher precision for 6 out of 7 ground truth-rarity datasets compared to those made only by PrimAlign. Importantly, the overlap between predictions of TARA++ and PrimAlign has much higher precision than either alone. This is not totally unexpected for reasons discussed in section “Discussion”.

*Running time.* We analyze the time needed for TARA-TS, TARA, and PrimAlign to compute an alignment when considering the ALL GO term rarity threshold; this threshold is the worst case (slowest) out of all studied thresholds since it has the most data. As TARA++ comes from the intersection of TARA-TS’s and TARA’s results, its time is either the maximum or sum of TARA-TS’s and TARA’s, if the two are run at the same time or one after the other, respectively. We find the following (also, see Additional file 1: Table S1).

As expected, we observe that TARA-TS’s running time decreases as *k* (in $\hbox {atleast}k$-EXP) increases, since there is less data overall, and thus less data to train on. When comparing TARA-TS and TARA, the former is faster, and this comes from the feature computation time, as both use the same supervised framework. TARA-TS’s node2vec computation is expectedly faster than TARA’s graphlet counting even when using Orca for two reasons. First, the random walks produced by node2vec can be thought of as sampling the network structure, which is much faster than capturing the full network structure like graphlets do.

Second, node2vec is parallelized while Orca is not. Parallelization benefits node2vec a lot: the same number of random walks is performed for each node (parameter -r:), so no single node takes much longer than any other. However, for graphlet counting, nodes with e.g., high degrees are the limiting time factor, and so parallelization would not help as much. Also note that TARA-TS’s (and PrimAlign’s) running time is missing the step of computing sequence-based anchor links; these anchors were precomputed and provided by the PrimAlign study. So, TARA-TS (and PrimAlign) has an unfair advantage over TARA. Despite this missing step, regardless of how TARA-TS and TARA are combined to form TARA++, PrimAlign will still be faster. However, it is about half as precise as TARA++. Even though TARA++ is slower, it is still practically feasible. Thus, the extra time is worth the almost doubling of precision.

*TARA++’s robustness to data noise.* Lastly, we investigate TARA++’s robustness to noise (i.e., random perturbation) in the data, since one cannot expect all real-world data (even the PPI networks we use!) to be perfect. When we incrementally introduce noise in the data, ranging from 0% (original data) to 100% (completely random), we find that TARA++ is fairly robust up to 50% noise (Fig. 8 and Additional file 1: Section S2.3). Beyond 50%, precision and recall drop and eventually reach 0, as expected.

## Discussion

Recall the two unexpected findings from section “Results – Comparison of TARA-TS versions”. Namely, first, it is surprising that TARA-TS (graphlets) does not improve upon TARA, i.e., that the additional sequence information does not improve upon only topological information. A reason may be that the across-network sequence information complements, rather than enhances, the within-network topology information. Some of the predictions made by TARA-TS (graphlets), specifically those that overlap with TARA’s, may be due to the within-network topology information used by both methods, and the remaining predictions made by TARA-TS (graphlets) may be due to the across-network sequence information, which is not used by TARA. Second, it is surprising that TARA-TS (metapath2vec) does not improve upon TARA-TS (node2vec). Both use a similar random walk-based embedding process, but metapath2vec additionally accounts for the heterogeneous information in the integrated network. The lack of improvement may be because the additional information captured by the considered metapaths is not useful in this task, or because constraining random walks by node type leads to less neighborhood structure being explored. For example, at some point in a random walk, a human node may have many human neighbors, but the walk is forced to move to a yeast node due to the metapath constraints. Then, the neighborhood of that human node will not be well explored. However, because the number of possible metapaths to test in order to find the best one(s) is exponential with respect to the length of the path, it is not feasible to test every possibility, even for short lengths. Thus, an efficient way of selecting appropriate metapaths for a given network would be necessary to continue to pursue metapath-based embedding methods for this task. However, to our knowledge no such selection process exists, which is why we do not pursue this problem beyond the metapaths we have considered.

Also recall the two interesting findings from section “Results – TARA-TS versus TARA in the task of protein functional prediction: toward TARA++”. Namely, first, graphlets, which use only topological information, perform as well as network embedding features that use both topological and sequence information. This motivates the need to develop better graphlet-based methods for integrated networks as future work. Second, predictions made by both TARA and TARA-TS are significantly more accurate than predictions made by any method alone. In a sense, their overlap is integrating state-of-the-art research across the computational biology and social network domains, by combining TARA’s graphlet-based topology-only features with TARA-TS’s embedding-based topology-and-sequence features. So, the overlapping predictions combine the strengths of both domains, showing promise for future domain-crossing endeavors.

Finally, recall from section “Results – TARA++ versus existing NA methods in the task of protein functional prediction” that the overlap between predictions of TARA++ and PrimAlign has much higher precision than either alone. This is not totally unexpected, as it suggests that predictions made by multiple methods (as already seen when combining TARA and TARA-TS into TARA++) are the most reliable; adding PrimAlign further strengthens this observation. Also, this echoes the promise of ensemble methods in machine learning. As such, further exploration of integrating different approaches beyond the simple overlapping of their predictions may be fruitful.

## Conclusions

TARA and TARA-TS are among the first *biological* NA methods that use supervised learning, despite the introduction of supervised *social* NA methods in recent years. This could be because the study of biological NA began well before the current era of “big data” [41, 42], making unsupervised approaches the traditional option. However, as the amount of biological network data continues to increase, developing data-driven approaches is an important direction. Especially fruitful for the task of NA is integrating research knowledge across biological and social network domains, as we have shown by combining TARA and TARA-TS into TARA++. Namely, TARA++ outperforms state-of-the-art NA methods in the task of protein functional prediction, an ultimate goal of NA. Though, it is still important to note that data-driven approaches are limited when data is scarce. As such, more sophisticated “ensembling” procedures for integrating protein functional prediction approaches together, beyond the simple overlapping of their predictions as we explored with TARA and TARA-TS (into TARA++), and PrimAlign, could potentially mitigate these limitations and open up new research directions.

As TARA++ is the first data-driven NA method to integrate topological and sequence information, it is just a proof-of-concept. This work can be taken further. We found that graphlet-based features on the isolated networks (on topological information alone) perform as well as embedding-based features on the integrated network (on topological and sequence information combined), even though the latter (using more data) was expected to be better. Thus, developing a graphlet feature that would efficiently deal with an integrated network could yield further improvements. This might include novel algorithms for speeding up counting of heterogeneous graphlets in large data. Heterogeneous graphlets, or heterogeneous network embedding features other than metapath2vec, could better distinguish between different node/edge types in an integrated network and thus only improve over the features considered in this study. Also, we focused on NA of static networks. However, research in NA of dynamic (e.g., aging- or disease progression-related) networks is becoming popular [43, 44]. So, our framework can be adapted to such novel NA categories.

## Supplementary Information

**Additional file 1.**

## Data Availability

The data and code used in this study are available at https://www.nd.edu/~cone/TARA++/.

## Abbreviations

NA: Network Alignment
TARA: DaTA-dRiven network Alignment
GO: Gene Ontology
PPI: Protein–Protein Interaction
TARA-TS: TARA-Topology and Sequence
GDV: Graphlet Degree Vector
