# Supplementary material for: Data-driven biological network alignment that uses topological, sequence, and functional information
Source: BMC Bioinformatics. 2021 Jan 29;22:34. doi: 10.1186/s12859-021-03971-6 (PMC7847157; doi:10.1186/s12859-021-03971-6)
Supplement: Supplementary file 1 — Additional file 1. [file 12859_2021_3971_MOESM1_ESM.pdf]

Supplementary material: Data-driven biological network alignment that uses topological, sequence, and functional information

Shawn Gu and Tijana Milenković
Department of Computer Science and Engineering, ECK Institute for Global Health,
Center for Network and Data Science,
University of Notre Dame, IN 46556.

E-mail: tmilenko@nd.edu

S1 Methods

S1.1 TARA-TS's feature extraction methodology

S1.1.1 Graphlets

TARA relies on graphlets, which are small subgraphs (a path, triangle, square, etc., generally up to five nodes). Graphlets are often used to summarize the extended neighborhood of a node into its feature vector, as follows. For a node, for each automorphism orbit (intuitively, node symmetry group) in a graphlet, one can count how many times the node participates in each graphlet at each of its orbits. Then, the graphlet-based feature vector of a node, or the node's *graphlet degree vector* (GDV), is formed out of the counts of all considered graphlets/orbits that appear in the node's extended network neighborhood; for details, see [6]. To obtain the feature of a node pair, TARA takes the element-wise absolute difference of the nodes' GDVs. We found this to be the best (i.e., most accurate) way to simultaneously combine GDVs of both nodes out of all ways that we tested [4]. So TARA-TS can apply the same graphlet counting procedure to the integrated network, obtaining the GDV for each yeast and human node, and taking the absolute difference of two nodes' GDVs to obtain the feature vector of the yeast-human node *pair*.

S1.1.2 Node2vec

Node2vec method uses random walks to explore the neighborhood of a node in a network. For a node u, random walks starting at u are performed, and the sequence of nodes visited by each random walk is recorded. The number of random walks performed per node is controlled by the parameter "Number of walks per source (`-r:`)", and the length of a random walk is controlled by the parameter "Length of walk per source (`-l:`)". This process is repeated for every node in the network. Then, a skip-gram model is applied over all sequences of nodes and the feature vector of each node is obtained; for details, see [2]. The only way to use node2vec, a single-network method, in the multi-network NA task, is to first integrate the two networks via anchor links, as we do. Otherwise, node2vec fails if applied to the two networks individually [3]. We first apply node2vec to the integrated network with the default parameters to obtain a feature vector for each *node*. Then, as suggested by the node2vec study [2], to get the feature vector of a yeast-human node *pair*, we take the element-wise average of the nodes' feature vectors.

We use node2vec over other network embedding methods for three reasons. (i) Even more recent methods, when evaluated in their own papers, achieve similar performance as node2vec in many tasks. So, we do not expect them to outperform node2vec in our task. (ii) The node2vec source code is available and well documented, unlike

for many other methods. (iii) The goal of this study is not to find the absolute best feature vector for supervised NA, but to test how combining topological and sequence information in supervised NA affects protein functional prediction. If using node2vec already improves upon current NA methods, then using any more sophisticated ways to extract features will only improve further. In our proposed framework, features from any new extraction method can simply be "swapped" in, allowing flexibility for further advancements.

S1.1.3 Metapath2vec

Metapath2vec requires the user to define "metapaths", which direct how the random walks move. A metapath example is "human-human-yeast-yeast" (or "human×2 → yeast×2"): start at a human node, move to a randomly chosen neighboring (RCN) human node, move to an RCN yeast node, and move to an RCN yeast node. This metapath is extended such that its length is as close as possible to the `-l:` parameter value. For example, if this value is 12, then this metapath would be repeated $\lfloor 12/4 \rfloor = 3$ times. Then, given a node u and the extended metapath, random walks starting at u are performed such that the nodes visited follow the constraints of the metapath, and the sequence of nodes visited by each random walk is recorded. In the process, node2vec's `-l:` and `-r:` parameters apply to metapath2vec as well. The procedure is repeated for every node in the network. Then, a skip-gram model is applied over all sequences of nodes to obtain node features. We use the metapath2vec++ implementation of metapath2vec [1] with the default parameters to obtain each node's feature vector, and again take the element-wise average of two nodes' feature vectors to compute the feature vector of a node *pair*. Choosing "optimal" metapaths is non-trivial and often the selection process involves using the same paths as those of previous studies [7, 1]. However, to our knowledge, this study is the first to investigate metapaths on an integrated across-species biological network. Thus, our only option is to do our due diligence and examine reasonable metapaths, to give a fighting chance to metapath2vec. We test these metapaths: "human×n → yeast×n" and "yeast×n → human×n" for $3 \leq n \leq 10$, "human×25 → yeast×25" and "yeast×25 → human×25", "human×50 → yeast×50" and "yeast×50 → human×50", and the combination of all of the individual metapaths (i.e., we apply the skip-gram model to all node sequences obtained from all considered metapaths). We have verified that the choice of metapath does not affect protein functional prediction accuracy, as illustrated in Supplementary Fig. S1 (see Sections "Methods – TARA-TS's classification and alignment generation" and "Methods – Using an alignment for protein functional prediction" for evaluation details). This may be because the metapaths we have chosen are all performing well, or because we have yet to find the best metapaths. Regardless, our goal is to test a variety of reasonable metapaths and choose the best out of them; finding optimal paths is beyond the scope of this study. Because the metapath choice does not impact accuracy in this case, for simplicity, we continue with the combination of all the metapaths.

S2 Results

S2.1 TARA-TS versus TARA in the classification context

Here, we comment on the performance of TARA-TS; recall that we use TARA-TS to refer to any of TARA-TS (graphlets, node2vec, metapath2vec). For a fixed GO term rarity threshold, as k in our atleast$k$-EXP ground truth datasets increases, we expect TARA-TS's (and TARA's) accuracy and AUROC to increase, as the condition for proteins to be functionally related becomes more stringent and thus the functional data becomes of higher quality. Also, for a fixed k, as we decrease the GO term rarity threshold (i.e., consider rarer GO terms), we expect accuracy and AUROC to increase, since rarer GO terms may be meaningful [5], again resulting in higher-quality data. We find the former expectation to hold, for all GO term rarity thresholds (Supplementary Figs. S1-S2). However, for the latter expectation, we find that classification accuracy and AUROC somewhat decrease (Supplementary Figs. S1-S2). This may be because as rarer GO terms are considered, the amount of training data decreases, which is what could be causing performance decreases.

As we increase y, the amount of training data, we expect accuracy and AUROC to increase, as more data is used during classification. For accuracy, for TARA-TS (graphlets), we observe this for 6/7 ground truth-rarity

datasets, although for 4/6 of the datasets, the increase is minimal (∼1%). In the remaining case, the accuracy increases until about 60% training data, and then drops. For TARA-TS (node2vec), we observe this for 6/7 ground truth-rarity datasets, although for 4/6, the increase is minimal. In the remaining case, the accuracy increases until about 60% training data, and then drops. For TARA-TS (metapath2vec), we observe this for all 7 ground-truth rarity datasets. For AUROC, for TARA-TS (graphlets), we observe the expected trend for all ground truth-rarity datasets, although for 4/7 of these datasets, the increase is minimal (∼1%). For both TARA-TS (node2vec) and TARA-TS (metapath2vec), we observe the expected trend for all ground-truth rarity datasets, although for 3/7 of these datasets, the increase is minimal. These unexpected trends (mostly minor increase of accuracy and AUROC even with large increase of y) are promising though, because they mean that TARA-TS does not have to use a majority of the functional data for training to still obtain good results; even using only 10% of the data seems to suffice.

S2.2 Matching the number of predictions made by TARA++

Here, we describe the process for ensuring the different methods make the same number of predictions as TARA++. For a given ground truth-rarity dataset, for a given method, we first rank the predicted protein-GO term associations based on their p-values in the hypergeometric tests (Section "Methods – Using an alignment for protein functional prediction"), where a smaller p-value means a better rank. Then, we take the top k predictions, where k is equal to the number of predictions made by TARA++. In this way, the best k predictions of each method are chosen, and every method makes the same number. Finally, we compute the precision and recall of those k predictions (Fig. 6 and Supplementary Fig. S12). In terms of precision, we find TARA++ is still the best in 4/7 cases. It is inferior to PrimAlign for 1/7 cases, and only slightly worse than TARA in 2/7 cases. In terms of recall, TARA++ is the best in 4/7 cases, tied with TARA in 1/7 cases, and slightly worse than TARA in 2/7 cases. Given these results, we believe that combining TARA and TARA-TS into TARA++ does generally lead to more reliable predictions than other methods, and that TARA++'s high precision is not simply due to it making fewer predictions. In a way, TARA-TS filters out the unreliable predictions from TARA and vice versa.

S2.3 Robustness of TARA++ to data noise

Here, we describe how we test TARA++'s robustness to data noise. We introduce three types of noise. (i) We randomly rewire x% of the PPI edges in each of the yeast and human networks. This means that for each network, we randomly delete x% of the edges and randomly add the same number of edges back such that there are no duplicates. (ii) We randomly rewire x% of the across-network anchor links. This means that we randomly delete x% of the yeast-human anchor links and randomly add the same number of yeast-human links back such that there are no duplicates. (iii) For a given ground truth-rarity dataset, we randomly rewire x% of the protein-GO term associations. This means that we randomly delete x% of the protein-GO term associations and randomly add the same number of associations back (out of all possible protein-GO term associations for the given ground truth-rarity dataset) such that there are no duplicates. We vary x from 0 to 100 in increments of 10. We apply each type of noise to its respective data, input the data into TARA and TARA-TS's frameworks, and combine their results into TARA++ as before. We do this for GO term rarity threshold 25 and ground truth dataset atleast2-EXP, as this is where TARA++ has the highest precision.

We find that TARA++'s precision actually increases until 50% noise, after which it then drops and eventually reaches 0, and its recall steadily decreases to 0 (Fig. 8). These trends are somewhat expected. As noise increases, the amount of meaningful information in the data decreases, resulting in fewer possible predictions. As such, recall naturally decreases. Because of these fewer predictions though, precision naturally increases. But at a certain point, the data is too noisy, so even the small number of predictions are mostly incorrect, and eventually no predictions can be made. Thus, at that point, precision decreases until it reaches 0.

	atleast1-EXP	atleast2-EXP	atleast3-EXP
TARA-TS	3811	480	444
TARA	8090	4676	4634
PrimAlign	16	16	16
Sequence	N/A	N/A	N/A

Supplementary Table S1: Running times (in seconds) of TARA-TS, TARA, PrimAlign, and Sequence, when considering ALL GO terms. TARA++'s running time is a function of TARA-TS's and TARA's (see Section "Results – TARA++ versus existing NA methods in the task of protein functional prediction" in the main paper). We use a precomputed alignment for Sequence (see Section "Results – TARA++ versus existing NA methods in the task of protein functional prediction" in the main paper), hence the "N/A"s.

S3 Supplementary figures and tables

References

[1] Y. Dong, N. V. Chawla, and A. Swami. metapath2vec: Scalable representation learning for heterogeneous networks. In *Proceedings of the 23rd ACM SIGKDD International Conference on Knowledge Discovery and Data Mining*, pages 135–144. ACM, 2017.

[2] A. Grover and J. Leskovec. node2vec: Scalable feature learning for networks. In *Proceedings of the 22nd ACM SIGKDD International Conference on Knowledge Discovery and Data Mining*, pages 855–864. ACM, 2016.

[3] S. Gu and T. Milenković. Graphlets versus node2vec and struc2vec in the task of network alignment. *In Proceedings of the 14th International Workshop on Mining and Learning with Graphs (MLG) at the 24th ACM SIGKDD International Conference on Knowledge Discovery and Data Mining*, 2018.

[4] S. Gu and T. Milenković. Data-driven network alignment. *PLOS ONE*, 15(7):e0234978, 2020.

[5] W. B. Hayes and N. Mamano. SANA NetGO: a combinatorial approach to using Gene Ontology (GO) terms to score network alignments. *Bioinformatics*, 34(8):1345–1352, 2017.

[6] T. Milenković and N. Pržulj. Uncovering biological network function via graphlet degree signatures. *Cancer Informatics*, 6:CIN–S680, 2008.

[7] J. Shang, M. Qu, J. Liu, L. M. Kaplan, J. Han, and J. Peng. Meta-path guided embedding for similarity search in large-scale heterogeneous information networks. *arXiv preprint arXiv:1610.09769*, 2016.

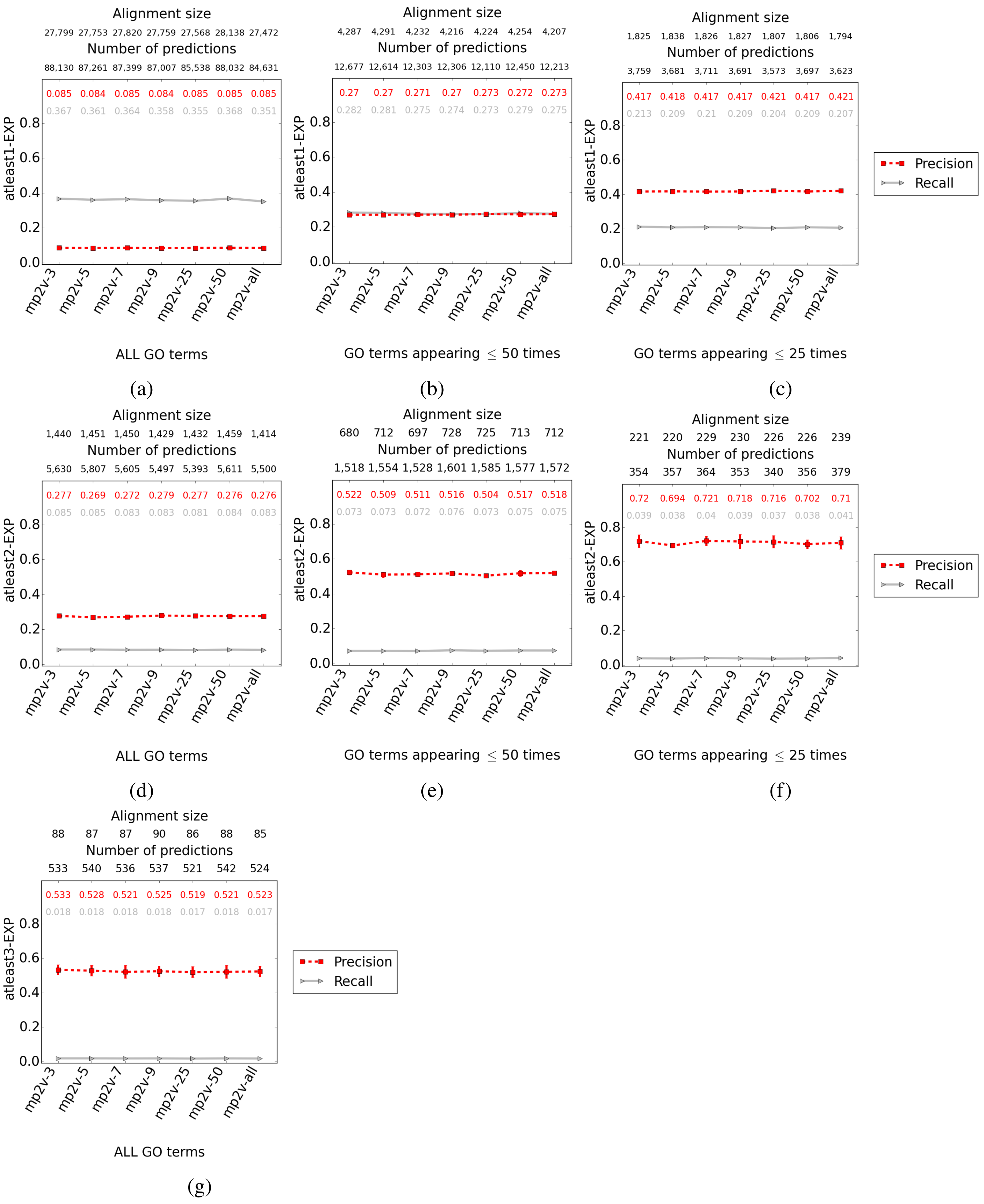

Supplementary Figure S1: Comparison of different metapath choices for rarity thresholds **(a, d, g)** ALL, **(b, e)** 50, and **(c, f)** 25 using ground truth datasets **(a, b, c)** atleast1-EXP, **(d, e, f)** atleast2-EXP, and **(g)** atleast3-EXP in the task of protein functional prediction. "mp2v-*n*" refers to the paths "human×n → yeast×n" and "yeast×n → human×n" (Section "Methods – TARA-TS's feature extraction methodology"). The alignment size (i.e., the number of aligned yeast-protein pairs) and number of functional predictions (i.e., predicted protein-GO term associations) made by each method are shown above. For example, the alignment for mp2v-3 in **(a)** contains 27,799 aligned yeast-human protein pairs, and predicts 88,130 protein-GO term associations. Raw precision and recall values are color-coded inside each panel.

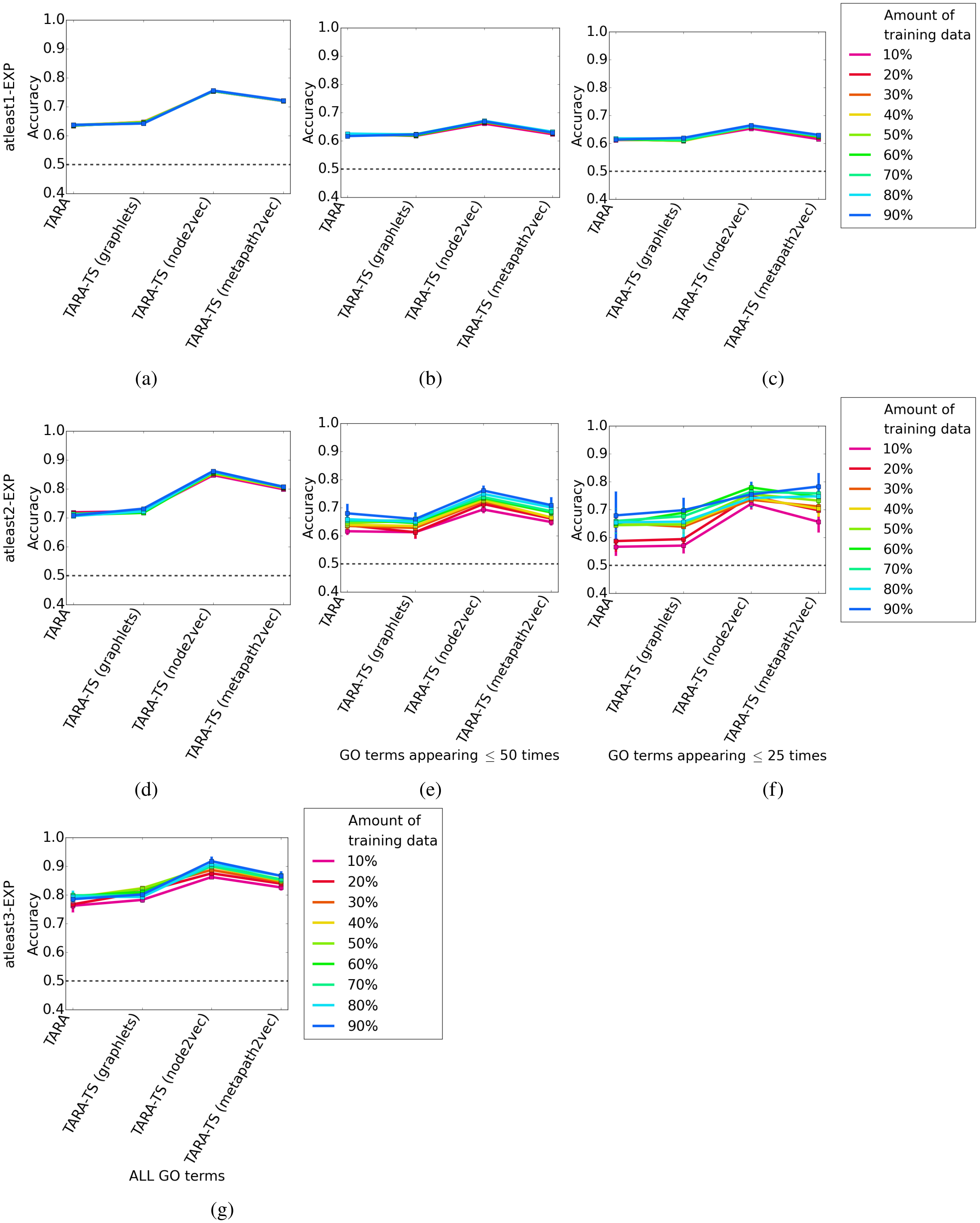

Supplementary Figure S2: Average prediction accuracy of percent training tests for rarity thresholds **(a, d, g)** ALL, **(b, e)** 50, and **(c, f)** 25 using ground truth datasets **(a, b, c)** atleast1-EXP, **(d, e, f)** atleast2-EXP, and **(g)** atleast3-EXP. A dotted black line indicates the accuracy expected if the classifier makes random predictions. Qualitatively similar results for AUROC are shown in Supplementary Figs. S2.

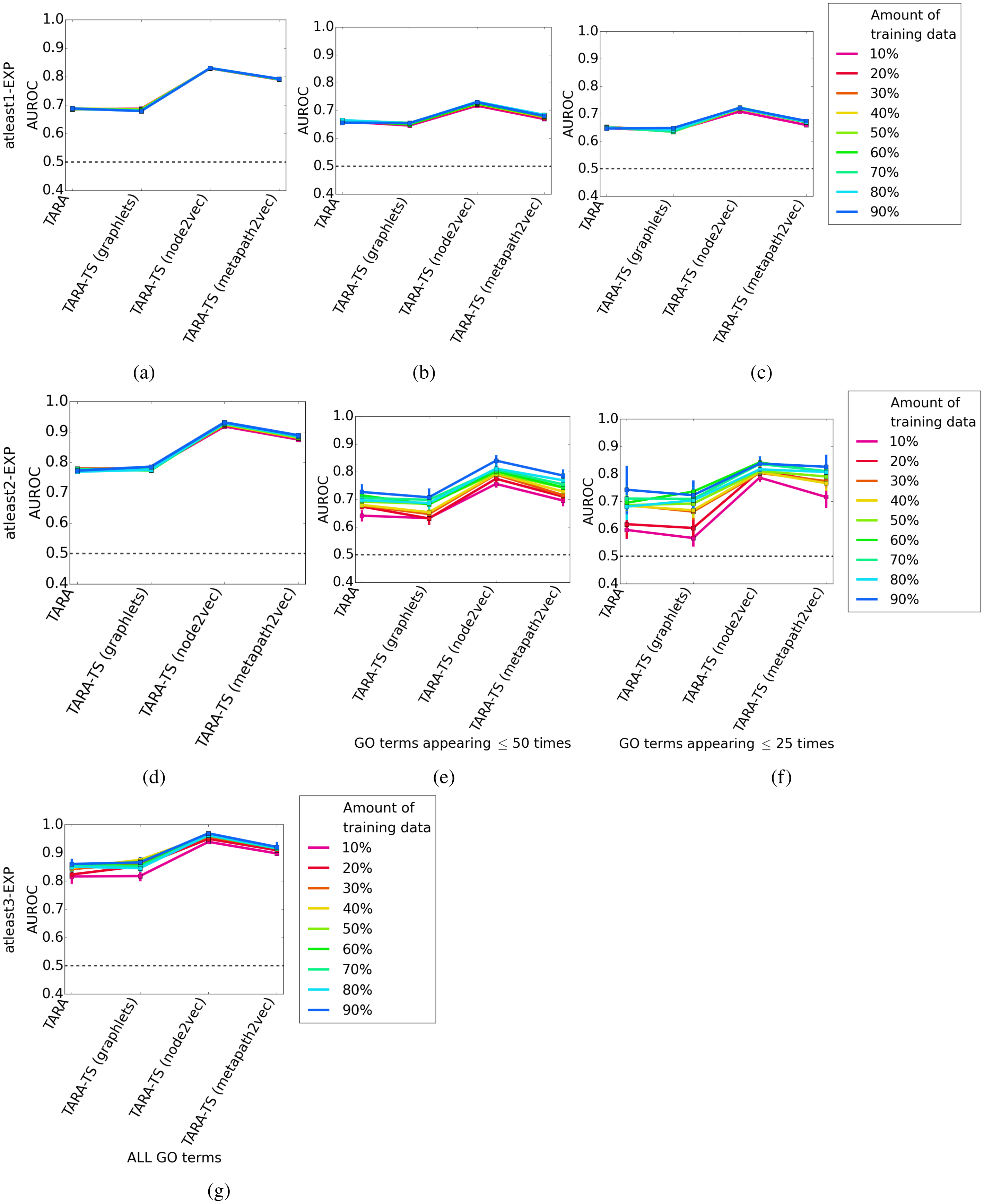

Supplementary Figure S3: Average AUROC of percent training tests for rarity thresholds **(a, d, g)** ALL, **(b, e)** 50, and **(c, f)** 25 using ground truth datasets **(a, b, c)** atleast1-EXP, **(d, e, f)** atleast2-EXP, and **(g)** atleast3-EXP. A dotted black line indicates the AUROC expected if the classifier makes random predictions.

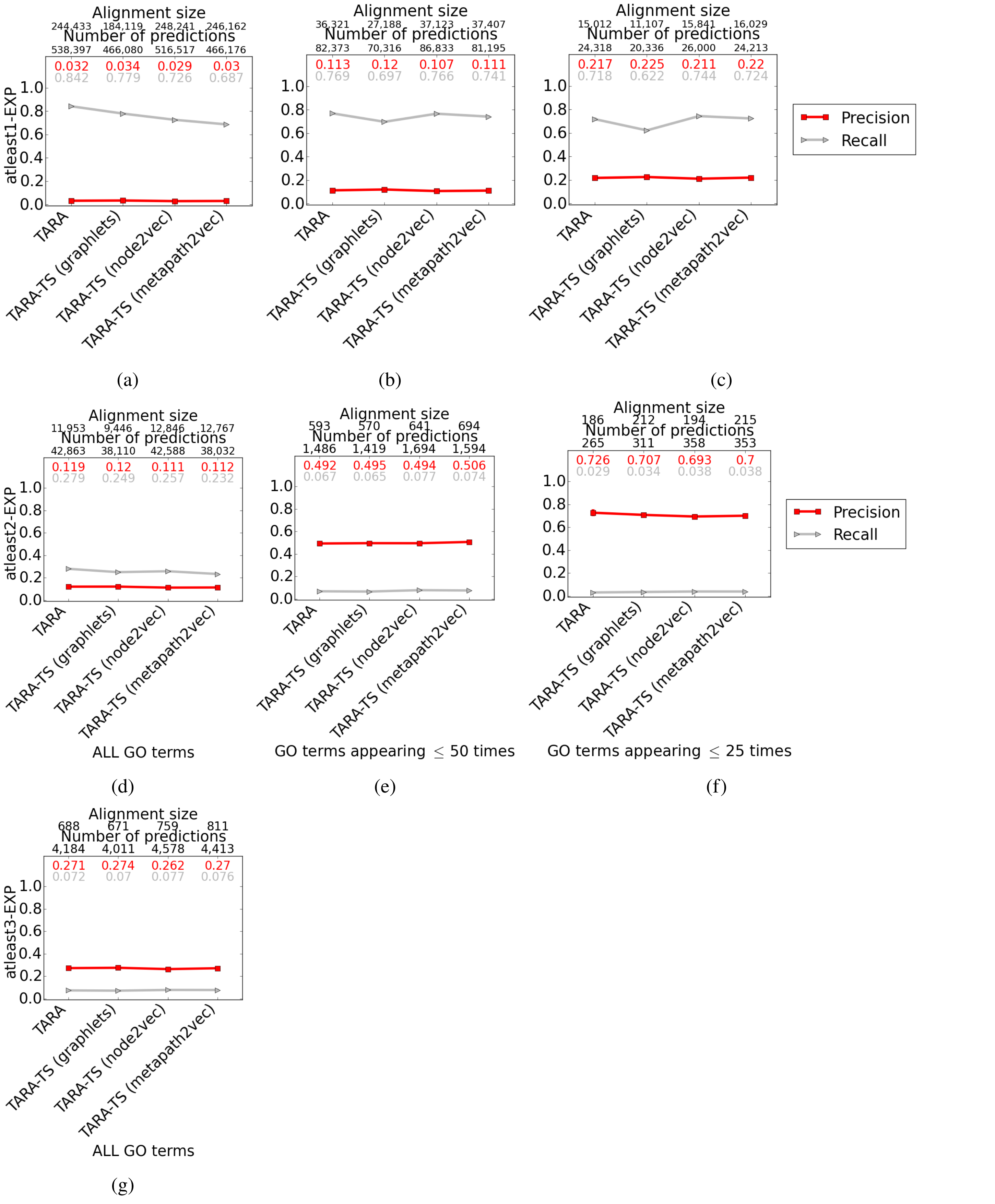

Supplementary Figure S4: Comparison of TARA and TARA-TS using 10% of the data as training for rarity thresholds **(a, d, g)** ALL, **(b, e)** 50, and **(c, f)** 25 using ground truth datasets **(a, b, c)** atleast1-EXP, **(d, e, f)** atleast2-EXP, and **(g)** atleast3-EXP in the task of protein functional prediction. The alignment size (i.e., the number of aligned yeast-protein pairs) and number of functional predictions (i.e., predicted protein-GO term associations) made by each method are shown above. For example, the alignment for TARA-10 in **(a)** contains 244,433 aligned yeast-human protein pairs, and predicts 538,397 protein-GO term associations. Raw precision and recall values are color-coded inside each panel.

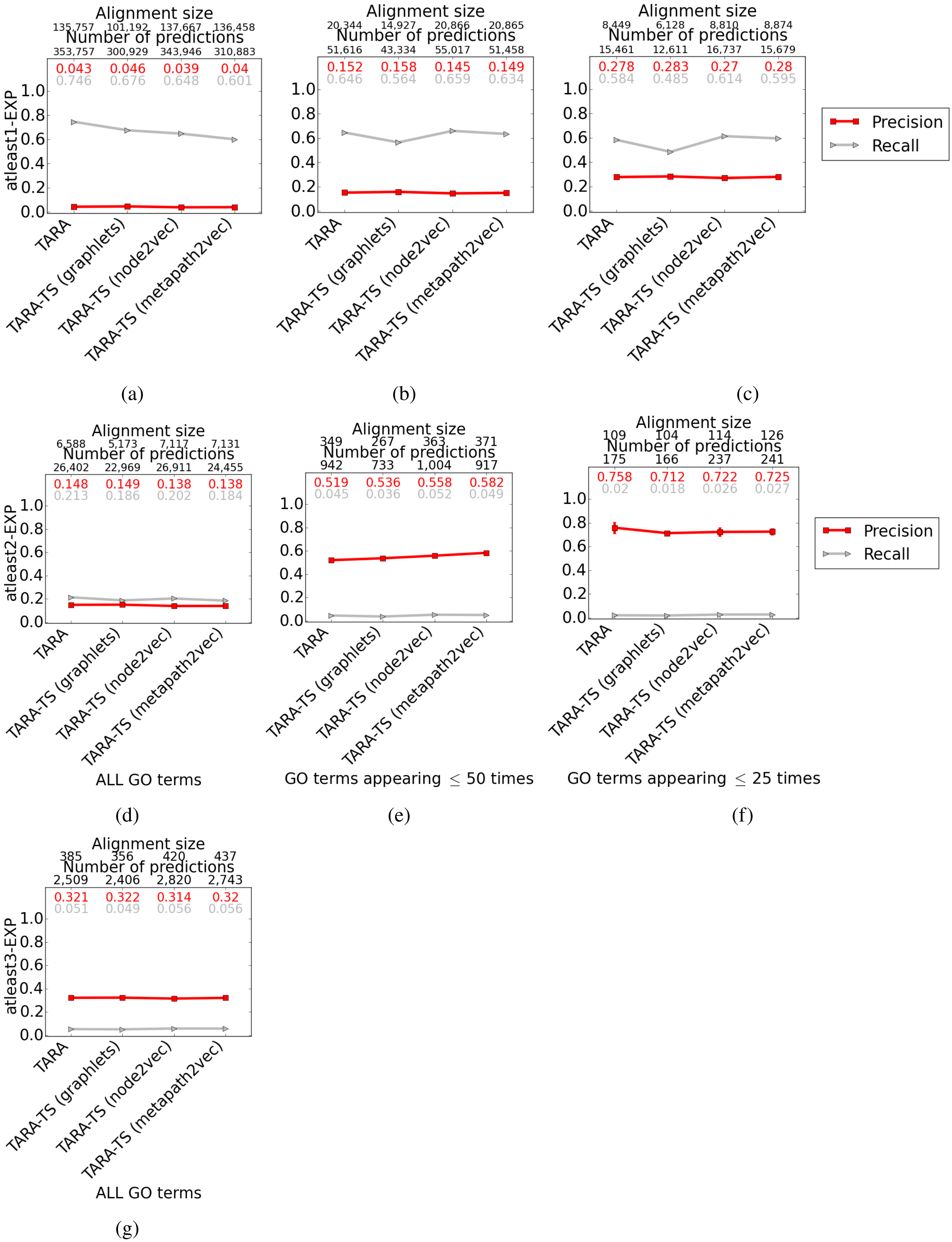

Supplementary Figure S5: Comparison of TARA and TARA-TS using 50% of the data as training for rarity thresholds **(a, d, g)** ALL, **(b, e)** 50, and **(c, f)** 25 using ground truth datasets **(a, b, c)** atleast1-EXP, **(d, e, f)** atleast2-EXP, and **(g)** atleast3-EXP in the task of protein functional prediction. The alignment size (i.e., the number of aligned yeast-protein pairs) and number of functional predictions (i.e., predicted protein-GO term associations) made by each method are shown above. For example, the alignment for TARA-10 in **(a)** contains 244,433 aligned yeast-human protein pairs, and predicts 538,397 protein-GO term associations. Raw precision and recall values are color-coded inside each panel.

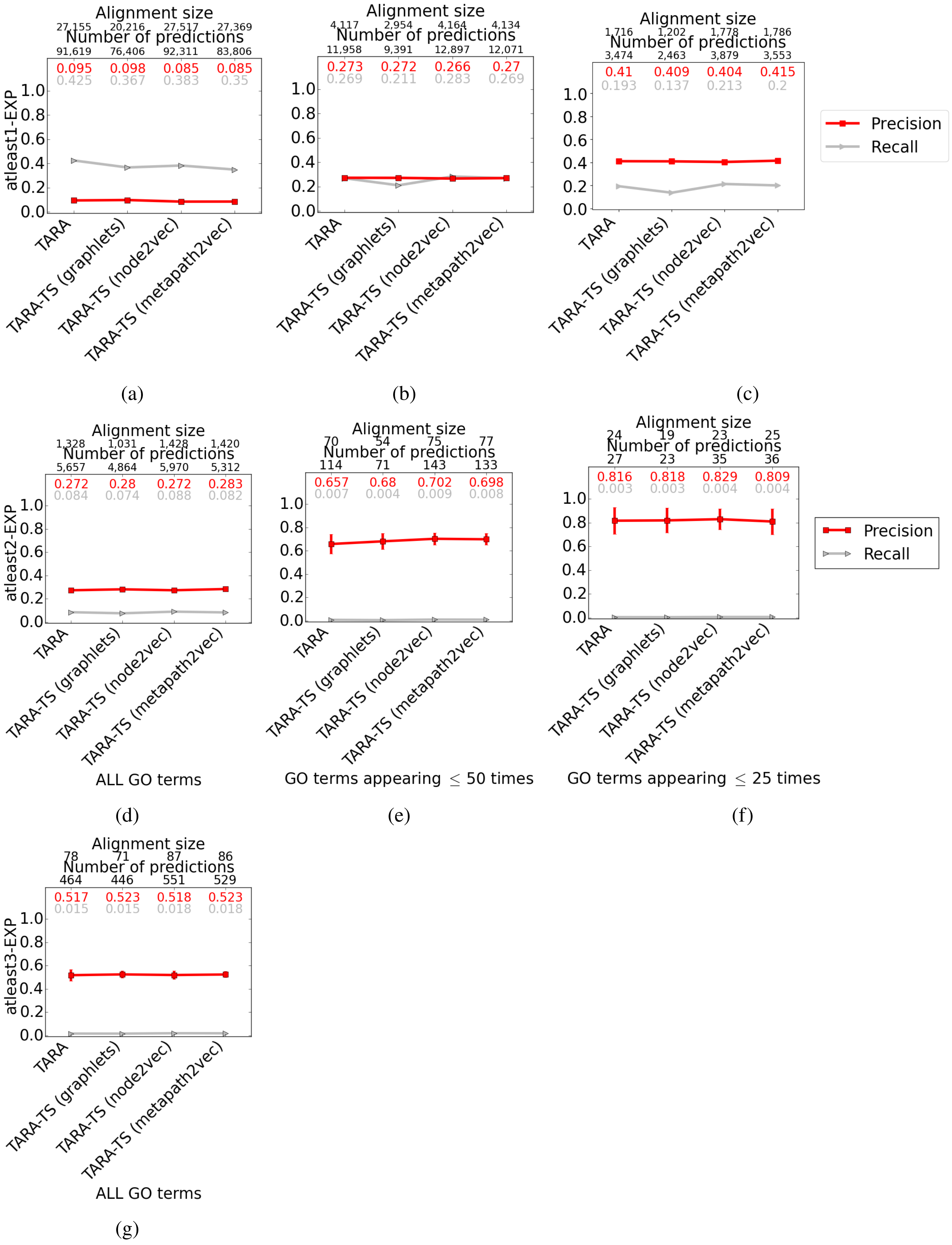

Supplementary Figure S6: Comparison of TARA and TARA-TS using 90% of the data as training for rarity thresholds **(a, d, g)** ALL, **(b, e)** 50, and **(c, f)** 25 using ground truth datasets **(a, b, c)** atleast1-EXP, **(d, e, f)** atleast2-EXP, and **(g)** atleast3-EXP in the task of protein functional prediction. The alignment size (i.e., the number of aligned yeast-protein pairs) and number of functional predictions (i.e., predicted protein-GO term associations) made by each method are shown above. For example, the alignment for TARA-10 in **(a)** contains 244,433 aligned yeast-human protein pairs, and predicts 538,397 protein-GO term associations. Raw precision and recall values are color-coded inside each panel.

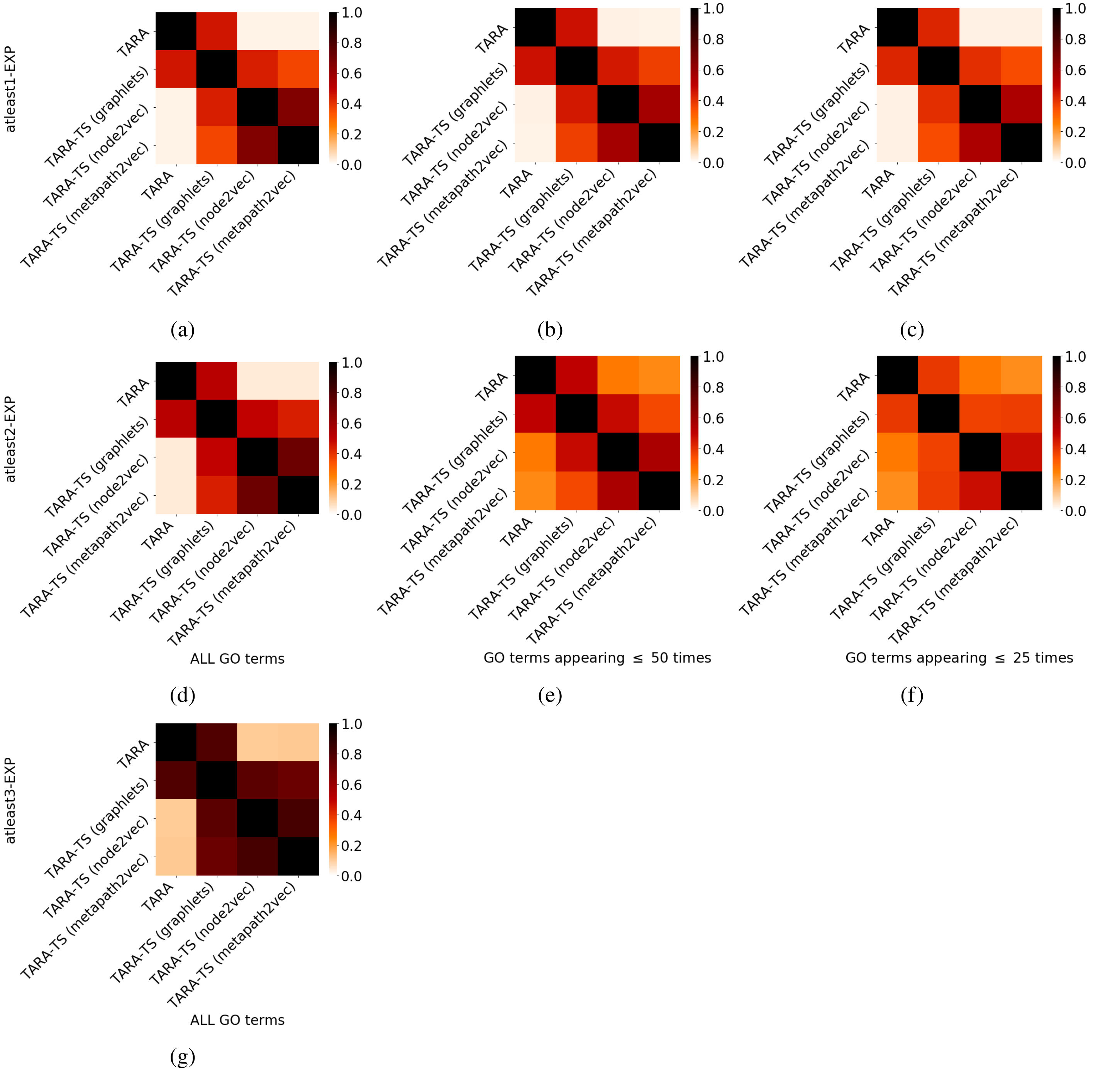

Supplementary Figure S7: Pairwise overlap, measured by Jaccard index, of the alignments made by TARA and TARA-TS for rarity thresholds **(a, d, g)** ALL, **(b, e)** 50, and **(c, f)** 25 using ground truth datasets **(a, b, c)** atleast1-EXP, **(d, e, f)** atleast2-EXP, and **(g)** atleast3-EXP, using percent training amounts described in Section "Results – TARA-TS versus TARA in the task of protein functional prediction: toward TARA++".

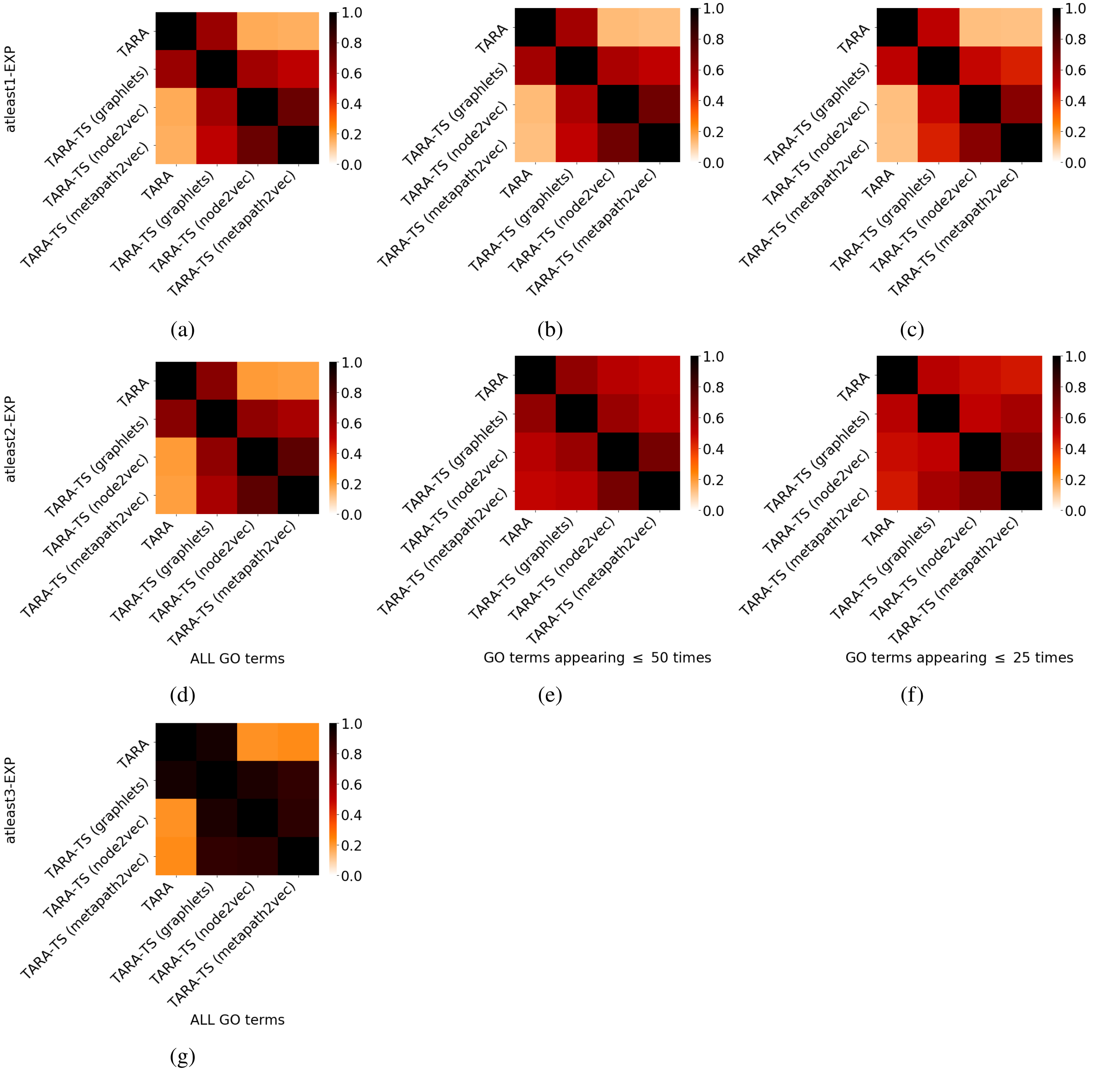

Supplementary Figure S8: Pairwise overlap, measure by Jaccard index, of the predictions made by TARA and TARA-TS for rarity thresholds **(a, d, g)** ALL, **(b, e)** 50, and **(c, f)** 25 using ground truth datasets **(a, b, c)** atleast1-EXP, **(d, e, f)** atleast2-EXP, and **(g)** atleast3-EXP, using percent training amounts described in Section “Results – TARA-TS versus TARA in the task of protein functional prediction: toward TARA++”.

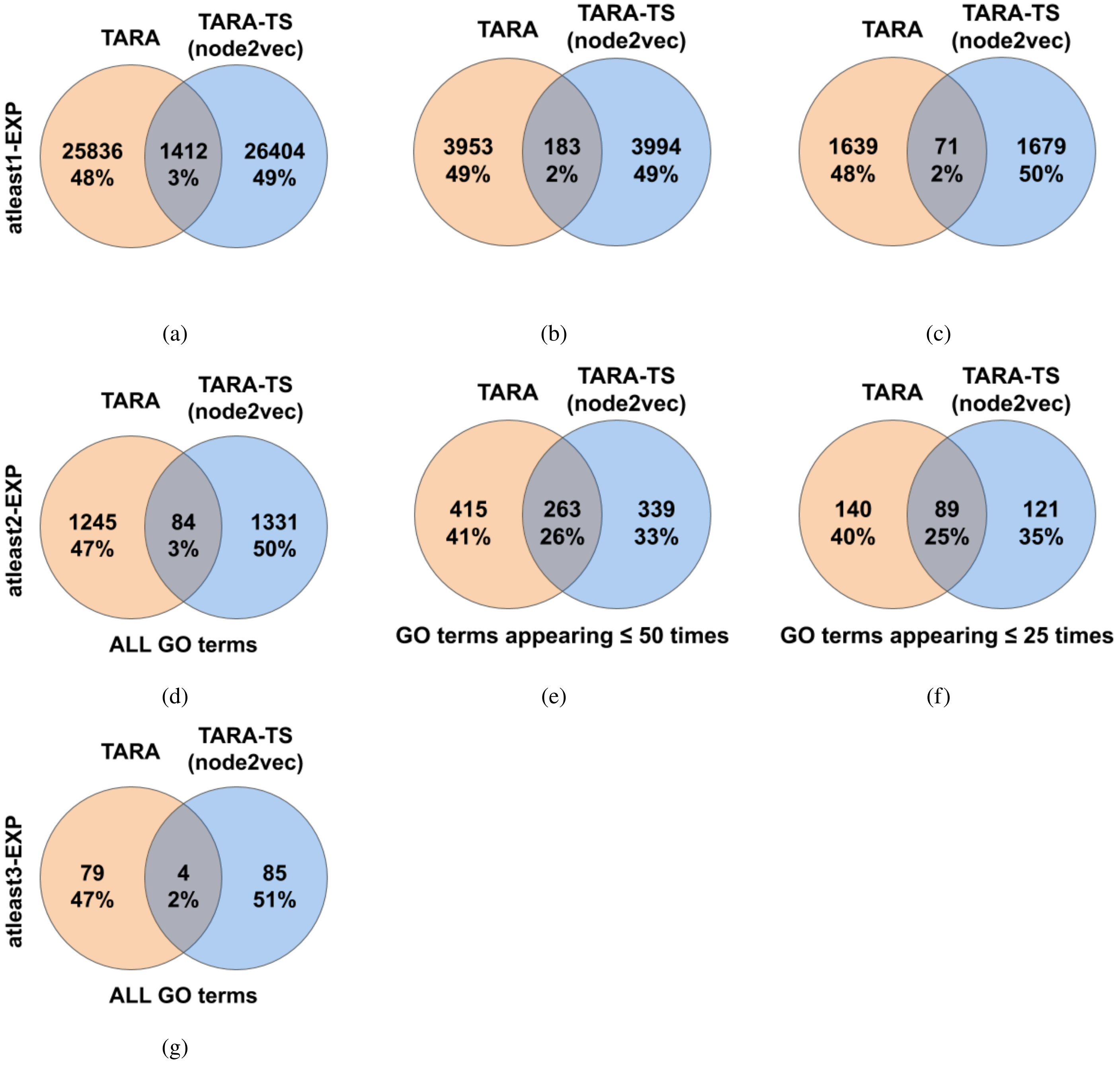

Supplementary Figure S9: Overlap of the alignments made by TARA and TARA-TS for rarity thresholds **(a, d, g)** ALL, **(b, e)** 50, and **(c, f)** 25 using ground truth datasets **(a, b, c)** atleast1-EXP, **(d, e, f)** atleast2-EXP, and **(g)** atleast3-EXP. Percentages are out of the total number of unique aligned node pairs made by both methods combined. The overlaps are for one of the 10 balanced datasets; so, the alignment size of a method may differ from those in Supplementary Figs. S3-S5, where the statistics are averaged over all balanced datasets.

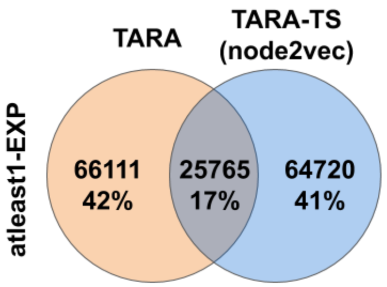

	TARA only	TARA++	TARA-TS only
Precision	0.047	0.22	0.032
Recall	0.15	0.28	0.10

(a)

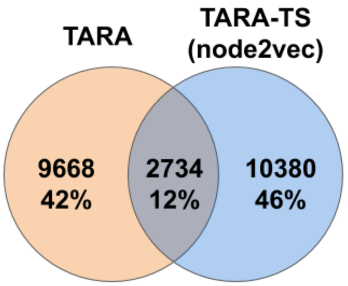

TARA only	TARA++	TARA-TS only
0.17	0.60	0.16
0.14	0.14	0.14

(b)

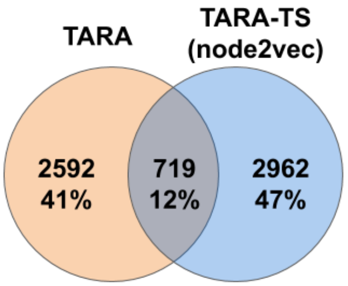

TARA only	TARA++	TARA-TS only
0.33	0.71	0.35
0.12	0.070	0.14

(c)

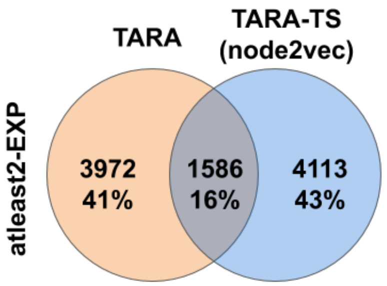

	TARA only	TARA++	TARA-TS only
Precision	0.18	0.54	0.18
Recall	0.038	0.047	0.038

(d)

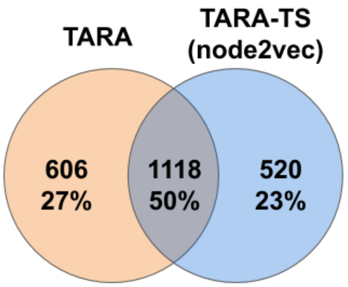

GO terms appearing ≤ 50 times

TARA only	TARA++	TARA-TS only
0.36	0.51	0.48
0.020	0.053	0.023

(e)

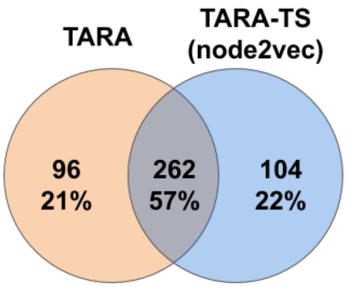

GO terms appearing ≤ 25 times

TARA only	TARA++	TARA-TS only
0.63	0.79	0.47
0.018	0.061	0.015

(f)

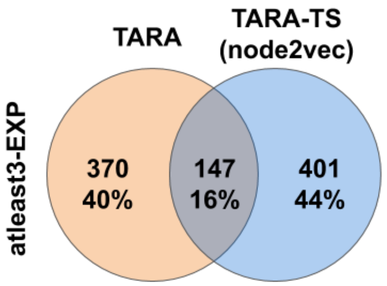

ALL GO terms

	TARA only	TARA++	TARA-TS only
Precision	0.41	0.78	0.40
Recall	0.0097	0.0073	0.010

(g)

Supplementary Figure S10: Overlap of the predictions made by TARA and TARA-TS for rarity thresholds **(a, d, g)** ALL, **(b, e)** 50, and **(c, f)** 25 using ground truth datasets **(a, b, c)** atleast1-EXP, **(d, e, f)** atleast2-EXP, and **(g)** atleast3-EXP. Percentages are out of the total number of unique predictions made by both methods combined. Precision and recall are shown for each of the three prediction sets captured by the Venn diagram; TARA++'s predictions are those in the overlap. The overlaps are for one of the 10 balanced datasets; so, the prediction number of a method may differ from those in Supplementary Figs. S3-S5, where the statistics are averaged over all balanced datasets.

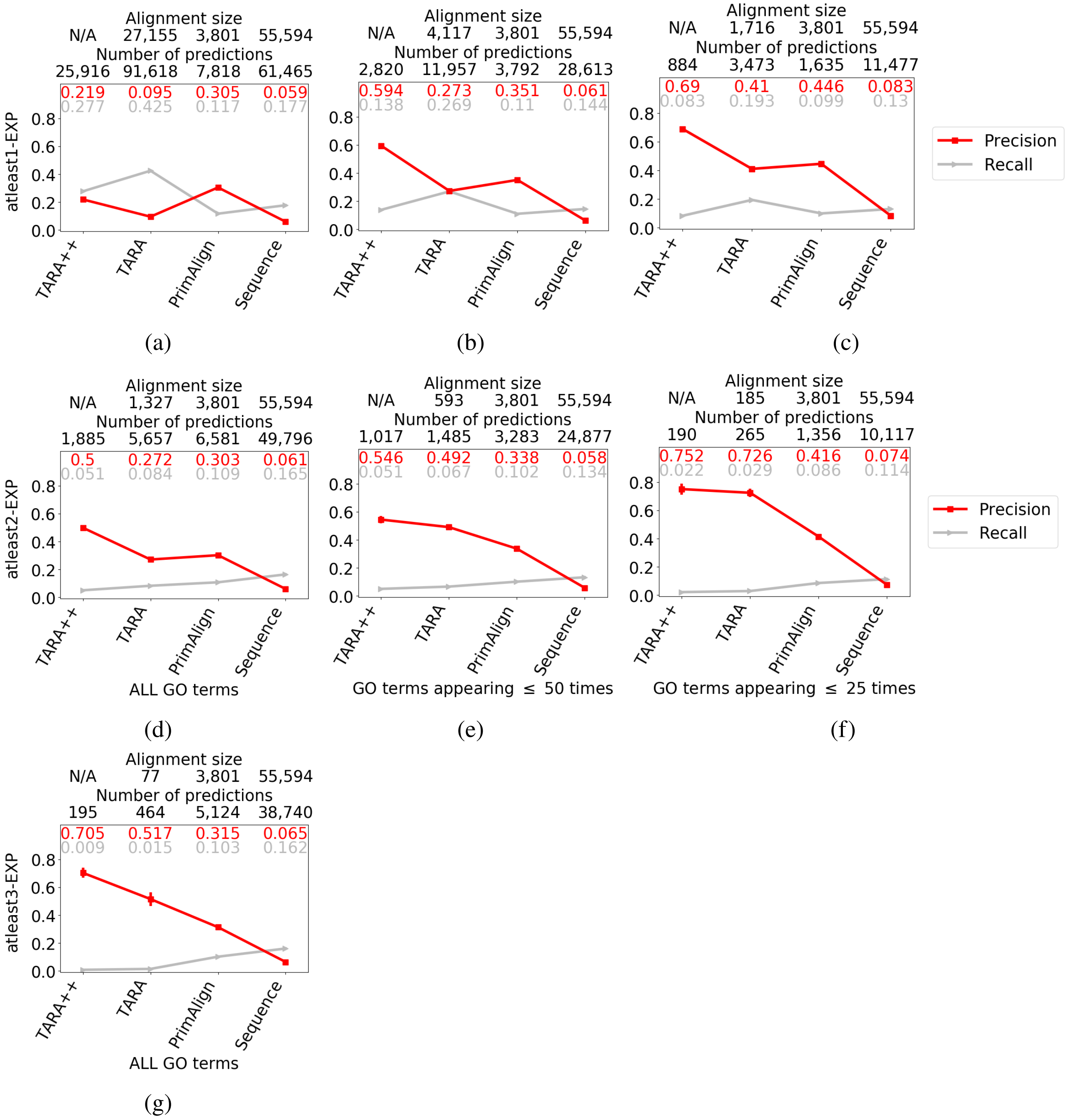

Supplementary Figure S11: Comparison of four NA methods for rarity thresholds **(a, d, g)** ALL, **(b, e)** 50, and **(c, f)** 25 using ground truth datasets **(a, b, c)** atleast1-EXP, **(d, e, f)** atleast2-EXP, and **(g)** atleast3-EXP in the task of protein functional prediction. The alignment size (i.e., the number of aligned yeast-protein pairs) and number of functional predictions (i.e., predicted protein-GO term associations) made by each method are shown above, except that TARA++ does not have an alignment *per se*. i.e., TARA++ comes from the overlap of *predictions* made by TARA and TARA-TS; hence the "N/A"s. For example, the alignment for TARA in **(a)** contains 27,155 aligned yeast-human protein pairs, and predicts 91,618 protein-GO term associations. Raw precision and recall values are color-coded inside each panel. For TARA++ and TARA, results are averages over all balanced datasets; the standard deviations are small and thus invisible.

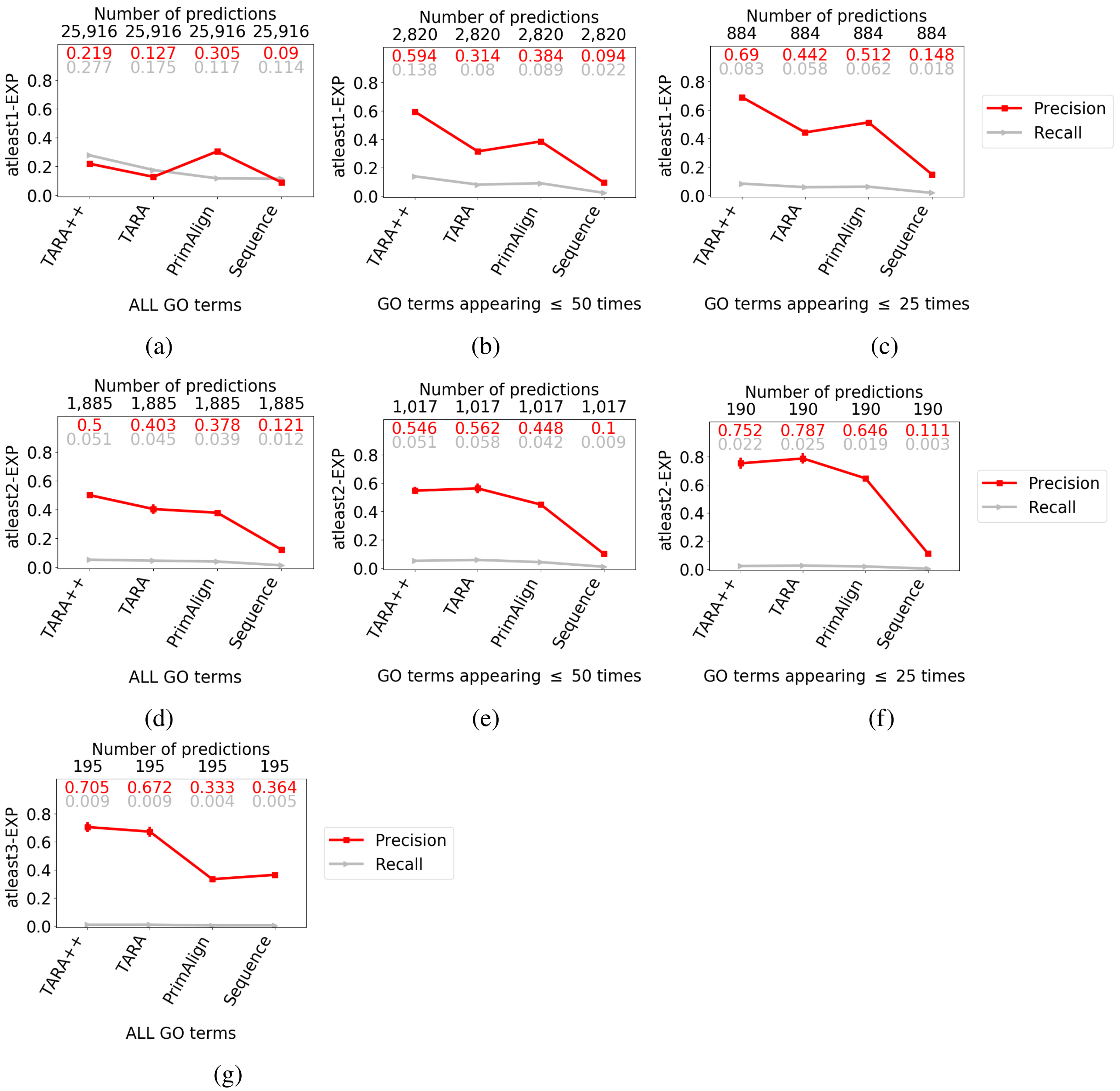

Supplementary Figure S12: Comparison of four NA methods for rarity thresholds **(a, d, g)** ALL, **(b, e)** 50, and **(c, f)** 25 using ground truth datasets **(a, b, c)** atleast1-EXP, **(d, e, f)** atleast2-EXP, and **(g)** atleast3-EXP in the task of protein functional prediction. The alignment size (i.e., the number of aligned yeast-protein pairs) and number of functional predictions (i.e., predicted protein-GO term associations) made by each method are shown above, except that TARA++ does not have an alignment *per se*. i.e., TARA++ comes from the overlap of *predictions* made by TARA and TARA-TS; hence the "N/A"s. For example, the alignment for TARA in **(a)** contains 27,155 aligned yeast-human protein pairs, and predicts 91,618 protein-GO term associations. Raw precision and recall values are color-coded inside each panel. For TARA++ and TARA, results are averages over all balanced datasets; the standard deviations are small and thus invisible.

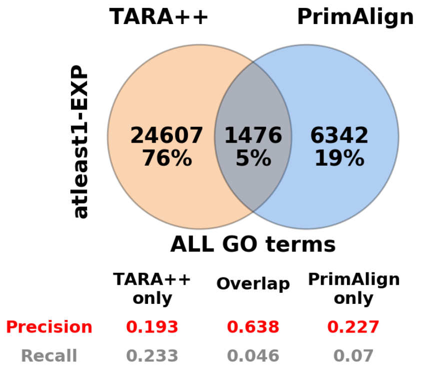

(a)

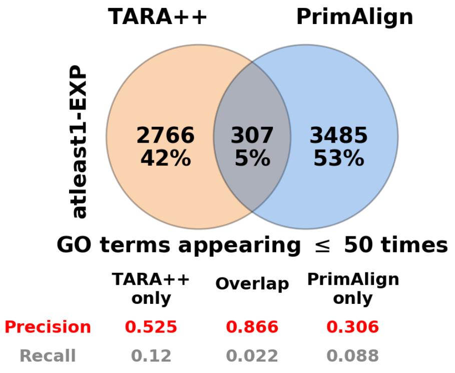

(b)

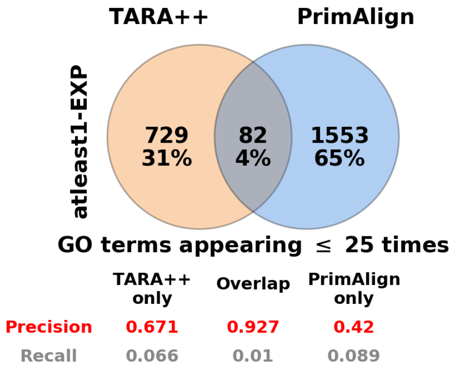

(c)

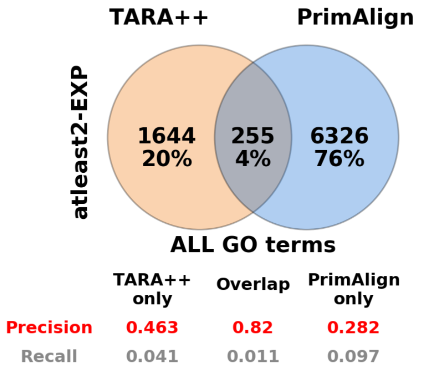

(d)

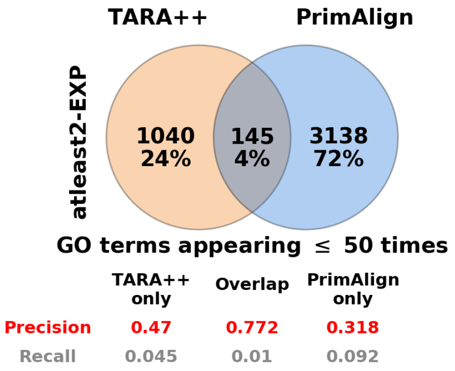

(e)

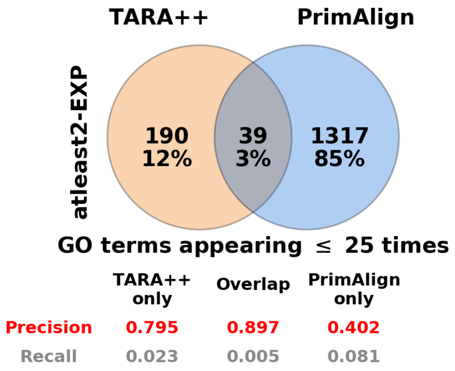

(f)

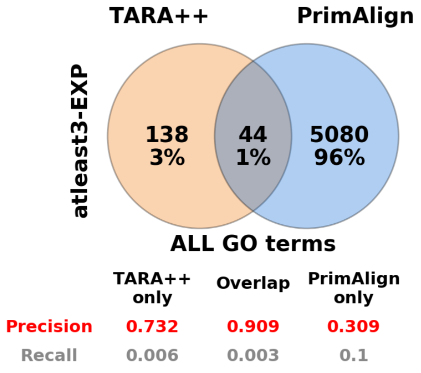

(g)

Supplementary Figure S13: Overlap of the predictions made by TARA++ and PrimAlign for rarity thresholds **(a, d, g)** ALL, **(b, e)** 50, and **(c, f)** 25 using ground truth datasets **(a, b, c)** atleast1-EXP, **(d, e, f)** atleast2-EXP, and **(g)** atleast3-EXP. Percentages are out of the total number of unique predictions made by both methods combined. Precision and recall are shown for each of the three prediction sets captured by the Venn diagram. The overlaps are for one of the 10 balanced datasets.
